# Nitrogen isotopes suggest a change in nitrogen dynamics between the Late Pleistocene and modern time in Yukon, Canada

**DOI:** 10.1371/journal.pone.0192713

**Published:** 2018-02-15

**Authors:** Farnoush Tahmasebi, Fred J. Longstaffe, Grant Zazula

**Affiliations:** 1 Department of Earth Sciences, The University of Western Ontario, London, Ontario, Canada; 2 Yukon Palaeontology Program, Department of Tourism & Culture, Government of Yukon, Whitehorse, Yukon Territory, Canada; Perot Museum of Nature and Science, UNITED STATES

## Abstract

A magnificent repository of Late Pleistocene terrestrial megafauna fossils is contained in ice-rich loess deposits of Alaska and Yukon, collectively eastern Beringia. The stable carbon (*δ*^13^C) and nitrogen (*δ*^15^N) isotope compositions of bone collagen from these fossils are routinely used to determine paleodiet and reconstruct the paleoecosystem. This approach requires consideration of changes in C- and N-isotope dynamics over time and their effects on the terrestrial vegetation isotopic baseline. To test for such changes between the Late Pleistocene and modern time, we compared *δ*^13^C and *δ*^15^N for vegetation and bone collagen and structural carbonate of some modern, Yukon, arctic ground squirrels with vegetation and bones from Late Pleistocene fossil arctic ground squirrel nests preserved in Yukon loess deposits. The isotopic discrimination between arctic ground squirrel bone collagen and their diet was measured using modern samples, as were isotopic changes during plant decomposition; Over-wintering decomposition of typical vegetation following senescence resulted in a minor change (~0–1 ‰) in *δ*^13^C of modern Yukon grasses. A major change (~2–10 ‰) in *δ*^15^N was measured for decomposing Yukon grasses thinly covered by loess. As expected, the collagen-diet C-isotope discrimination measured for modern samples confirms that modern vegetation *δ*^13^C is a suitable proxy for the Late Pleistocene vegetation in Yukon Territory, after correction for the Suess effect. The N-isotope composition of vegetation from the fossil arctic ground squirrel nests, however, is determined to be ~2.8 ‰ higher than modern grasslands in the region, after correction for decomposition effects. This result suggests a change in N dynamics in this region between the Late Pleistocene and modern time.

## 1 Introduction

The terminal Pleistocene ~13,000 years ago was a time of dynamic changes in large mammal communities [1], climate [2], ice sheet extent [3], and range and composition of vegetation [4], all of which was accompanied by a large global rise in atmospheric CO_2_ concentration (*p*CO_2_) [5]. Given the strong feedback mechanisms among herbivores, plant nutrient contents and ecosystem nutrient cycling [6], a comparable shift in nutrient dynamics likely accompanied such major environmental changes. Faith [7] suggested that a mode transition in N cycling was the main cause of megafauna extinction in North America after the terminal Pleistocene, driven mainly by a change in the N content of plants. He argued that environmental changes including rising atmospheric CO_2_ concentrations and possibly elevated temperature and precipitation amounts shifted the nutrient cycle from an accelerating to a decelerating mode. In the accelerating mode, abundant, excess plant N was returned to the soil by herbivores in readily bioavailable forms. By comparison, the decelerating mode was associated with lower plant N contents–a feedback reflecting lower soil N contents arising in large part by reduced returns of easily bioavailable nitrogen to the soil via herbivore excreta. Consequences of this shift included lower plant and soil N contents, reduced rates of nutrient cycling through the food web, reduced forage production, a lower biomass carrying capacity of the ecosystem, and ultimately collapse of megafauna populations. A study of lake sediment *δ*^15^N from a wide range of ecosystems also reported a gradual decrease in N availability of terrestrial ecosystems between ~15,000 to 7,000 years ago, which suggests a shift in the nature of terrestrial N cycling [8].

A change in N dynamics and availability should be traceable using the *δ*^15^N of plants [9] and animals. Higher plant *δ*^15^N generally reflects higher N availability and a more open N cycle [10]. This higher *δ*^15^N is passed on to the second trophic level (consumers) through the food chain [11]. Several studies have reported significantly different *δ*^15^N for herbivores over different Quaternary time periods (pre-, full- and post-Last Glacial Maximum (LGM)) in Alaska [12] and Eurasia [13–18], and some related those differences to a possible shift in the *δ*^15^N of herbivore diet in response to climate change. Considering these studies and empirical evidence for the influence of environmental factors on terrestrial N dynamics [19–21], some changes in N-isotope dynamics should be expected between the Late Pleistocene and modern time. If this prediction is accurate, then a suitably calibrated N-isotope baseline for vegetation should be utilized for Late Pleistocene ecosystems when comparing the *δ*^15^N of fossil bone collagen with modern counterparts [22].

In this study, we combine the stable carbon- and nitrogen-isotope compositions of modern [9] and fossil plants and animals to test for changes in N-isotope dynamics in Yukon Territory, northwest North America, between the Late Pleistocene and present time. Our study was focused in the Yukon, including portions that were not glaciated during the Pleistocene. This unglaciated region, known as Beringia, was an important terrestrial biotic refugium. It was home to a large community of flora and megafauna, and was a migration route for animals and people between Asia and North America during fully glacial times [23, 24]. At the end of the Pleistocene, significant changes in soil, plant and animal communities greatly affected the face of Beringia [4, 25–28].

We measured the stable carbon- and nitrogen-isotope compositions of modern Yukon arctic ground squirrel (*Urocitellus parryii*) bone collagen and structural carbonate, and compared these results to isotopic data for modern Yukon plants collected earlier by Tahmasebi et al. [9]. From this comparison, we have established the C- and N-isotope discrimination between arctic ground squirrel bone collagen and diet. We also determined the post-senescence, over-winter changes in *δ*^13^C and *δ*^15^N of six species of the most common Beringian plants. We then compared these results with those for an archive of Late Pleistocene flora contained in permafrost-preserved nests of ancient arctic ground squirrels (*Urocitellus parryi*). This allowed us to determine the effects of decomposition on the isotopic composition of fossil plants recovered from fossil nests. We used the *δ*^13^C and *δ*^15^N of these plant macrofossils and fossil bones to establish the differences between modern and Late Pleistocene C- and N-isotope baselines for vegetation in this portion of eastern Beringia.

### 1.1 Terrestrial N cycling and plant *δ*^15^N

A higher rate of N loss processes (denitrification and volatilization) relative to internal N cycling (nitrification, plant uptake, mineralization and immobilization) produces a more open N cycle [29, 30]. The increased N supply for N loss reactions, and associated large isotopic fractionations, leaves the soil system enriched in ^15^N [31–33]. Higher plant *δ*^15^N reflects higher N availability and a more open N cycle in terrestrial ecosystems [8, 10], and provides an index of N availability [20]. The main differences between more and less open N cycles are illustrated in Supporting Information S1 Fig [9].

### 1.2 Plant and soil *δ*^13^C

Plant *δ*^13^C is a function of photosynthetic pathway (C_3_, C_4_, CAM) and environmental factors including CO_2_ source, *p*CO_2_, water availability, latitude, altitude and irradiation [34, 35]. Generally, plant *δ*^13^C is affected by changing the *δ*^13^C of source CO_2_ or by modifying the ratio of intercellular to ambient *p*CO_2_ (C_i_/C_a_) [36]. Wooller et al.’s [37] study of modern grasses and sedges from Alaska and Yukon Territory reported higher *δ*^13^C for plants from dry habitats than wet habitats. Plants respond to water stress through stomatal closure, which results in reduced discrimination again ^13^C [34].

A decrease in stomatal density and plant *δ*^13^C during the deglacial period of the Late Pleistocene (~15,000–12,000 years ago), when *p*CO_2_ increased from 190 to 280 ppm, has been reported for fossil limber pine needles from western USA rat middens [38]. This effect may have been further amplified since the Industrial Revolution (~ AD 1850) by an additional increase in *p*CO_2_ and decrease in *δ*^13^C_atm_ resulting from anthropogenic activities (known as the Suess effect) [39]. During the LGM *δ*^13^C_atm_ was ‒6.4 ‰ but has decreased steadily since the Industrial Revolution to ‒8.6 ‰ in AD 2013 [40].

Vegetation following different photosynthesis pathways imparts different *δ*^13^C signals to organic carbon (OC) transferred to sediments and soils [41], and this signal can be used to track vegetation changes [42, 43]. Decomposition, however, can cause changes in original OC-*δ*^13^C, as discussed below.

### 1.3 Bone *δ*^15^N and *δ*^13^C

Bone collagen is one of the most common tissues analyzed in trophic ecology and paleodietary reconstruction. The most widely observed isotopic discriminations between collagen and diet range from +3 to +6 ‰ for C (*Δ*^13^C_Col-diet_) [44, 45] and +2 to +5 ‰ for N (*Δ*^15^N_Col-diet_) [46–48]. The ^13^C-enrichment (+9 to +11 ‰) reported for rodents from diet to bioapatite structural carbonate [49, 50] can also be used for dietary reconstruction. The C-isotope spacing between structural carbonate and collagen (*Δ*^13^C_Sc-Col_) decreases with increasing trophic level [51] with a mean *Δ*^13^C_Sc-Col_ of +6 to +7 ‰ for herbivores, +5 ‰ for omnivores and +4 ‰ for carnivores [45]. This change may reflect different macromolecular compositions of diet among animals and/or different digestive physiologies of animals at different trophic levels [52].

### 1.4 Changes in *δ*^15^N and *δ*^13^C during plant decomposition

Several studies have reported ^13^C- and ^15^N-enrichment of decomposed plants, resulting in soil and sediment organic matter (OM) with higher *δ*^13^C and *δ*^15^N than fresh plant inputs (e.g. [53–56]). Possible causes include (i) kinetic isotopic fractionation during microbial respiration for C [57] and microbial metabolism for N, which results in contribution of ^13^C- and ^15^N-enriched microbial biomass to residual OM, and (ii) release of ^13^C-depleted CO_2_ during decomposition [57–59].

Microorganisms typically have higher *δ*^13^C (by 1–3 ‰) than fresh plants [58, 60, 61]. A key role for microbes in plant decomposition is supported by a general decrease in C/N for OM, approaching that of microbes [62], and an increased abundance of microbially derived compounds [63] with increasing soil depth. Enrichment in ^15^N of decomposed OM also has been explained by the higher *δ*^15^N of microbial products relative to fresh plant tissues; a metabolism-related positive trophic shift in *δ*^15^N (from 1.5 to 6 ‰) has been reported for soil microorganisms [58, 61]. Along with changes in isotopic composition, a decrease in total mass and C/N, and an increase in N content of plant detritus and soil OM, have been observed in most studies of decomposition under both aerobic and anaerobic conditions [64–67].

The permafrost-preserved ancient arctic ground squirrel nests, which are the focus of this study, are a mixture of plant remains, fungal hyphae, fecal pellets, seeds, hairs, insects and faunal remains [68]. Because of their originally high content of fresh OC, the nests can be considered as hot spots for microbial activity. Plant decomposition may occur on the land surface prior to gathering by squirrels, after collection and during storage in the active nest [69], and following burial and incorporation into the permafrost. Plant decomposition was likely very limited once in the permafrost zone, but probably not halted completely, given the possibility of sub-zero adapted microbes [70].

### 1.5 Study area

**Fig 1:**
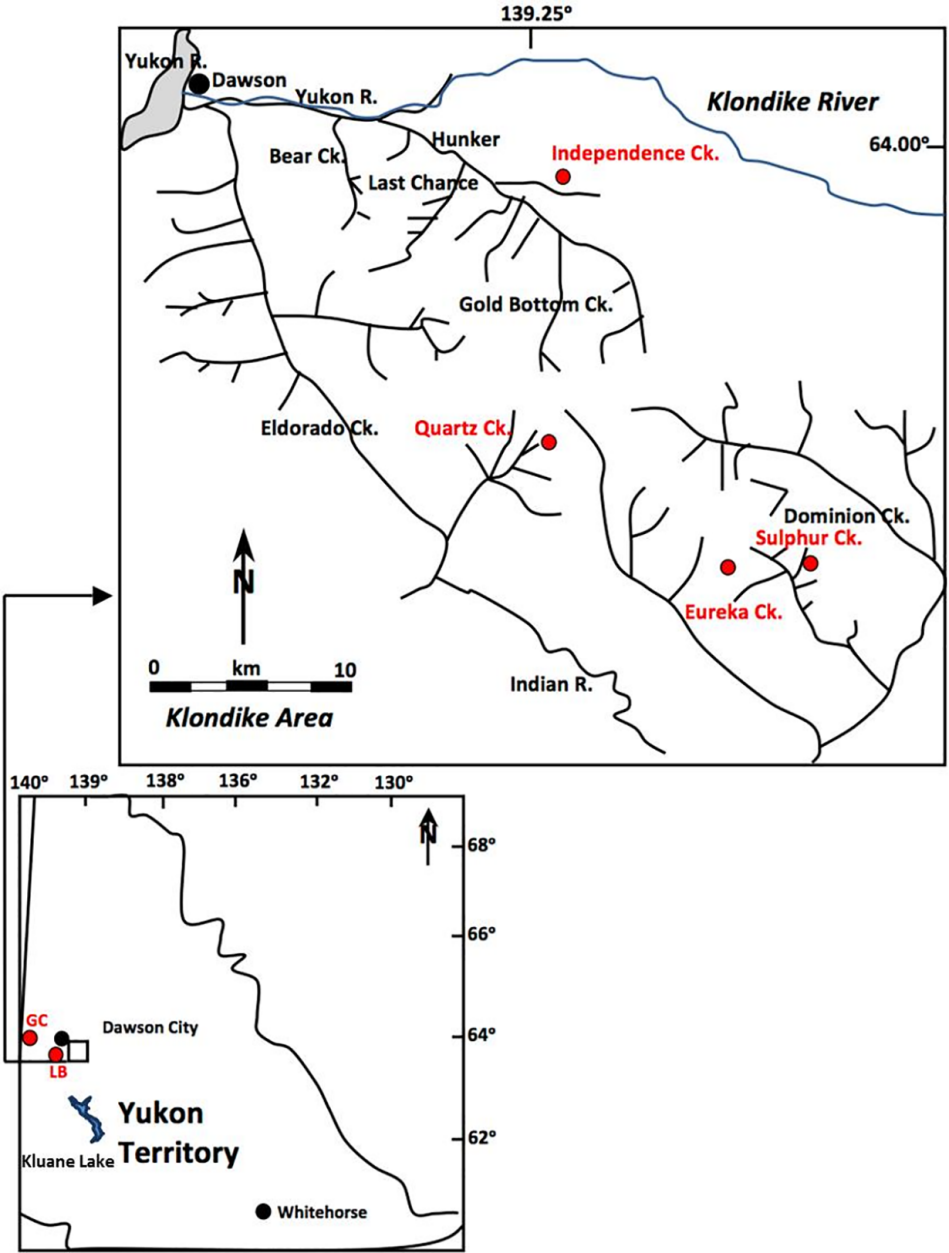
Sample locations. GC = Glacier Creek; LB = Little Blanche Creek. Adapted after Kotler and Burn [77].

The Klondike area of west-central Yukon Territory, Canada, is part of the unglaciated interior regions of Alaska and Yukon that comprise eastern Beringia. Placer gold mining in the Klondike area has exposed perennially frozen ice and organic-rich loess deposits that contain a wealth of information about Pleistocene ecosystems [71, 72]. The majority of loess and colluviated loess was deposited within valley bottom after 27ka BP followed by an accumulation of peaty organic material in the early Holocene [73]. The loess was likely derived from a combination of the floodplains of the Yukon and Klondike Rivers and local creek sources during Late Pleistocene dry periods [73]. Paleosols showing evidence of mild chemical weathering are preserved within these frozen sediments [74]. The loess deposits generally overlay gold-bearing valley bottom gravels. The loess deposits are rich in Pleistocene vertebrate remains, dominated by megaherbivores such as steppe bison (*Bison priscus*), woolly mammoth (*Mammuthus primigenius*) and horse (*Equus* spp.) [71]. Permafrost-preserved nests of ice age arctic ground squirrels from the loess deposits provide detailed floristic data on the paleoenvironment during cold and dry phases of Late Pleistocene glacial periods [68, 75, 76]. For the current study, we examined ancient arctic ground squirrel nests collected from placer gold mines at Quartz Creek (QC), Independence Creek (IC), Sulphur Creek (SC) and Eureka Creek (EC) (Fig 1). A few samples were also obtained from Glacier Creek (GC) in the Sixty Mile River area and Little Blanche Creek (LB) (Fig 1).

## 2 Materials and methods

### 2.1 Sampling

Thirty-six (36) fossil arctic ground squirrel nests were collected, 24 from two main sites QC and IC in summer 2013, and 12 from these and other sites prior to 2013 and maintained in frozen condition in the collections of the Yukon Palaeontology Program, Department of Tourism and Culture (Table 1). Eight loess samples were collected in summer 2013 at sites QC and IC. Sample collection and field work did not involve any endangered or protected species. The fossil bones collected were fully consumed by the isotopic analyses. Remaining fossil plant material is being utilized in related a biomarker investigation. Unused loess is stored at the University of Western Ontario. It will be returned to Yukon Territory in accordance with our research permit once all investigations are completed.

**Table 1:** General data for sampling sites.

Site	Site	Latitude	Longitude	Year of	# Nest	# Loess	ka BP^a^
ID	Location			Sampling	Samples	Samples	
**QC**	**Quartz Creek**	63.7513	‒139.1252	2012/2013	11	7	>49.9
**IC**	**Independence Creek**	63.9831	‒139.0212	2013	16	1	>41.2 to 22.52
**LB**	**Little Blanche Creek**	63.8312	‒139.0872	2009	1	-	>40.3
**EC**	**Eureka Creek**	63.6300	‒138.8251	2011	2	-	26.53
**GC**	**Glacier Creek**^**b**^	64.0368	‒140.8195	2013	1	-	16.58
**SC**	**Sulphur Creek**^**c**^	63.6480	‒138.6710	2011	5	-	21.18

^a^ Site dates are based on the oldest and youngest radiocarbon dates obtained at each location (details in S1 Table).

^b^ Sixty Mile River area.

^c^ Dan Klipper/Rod Smith Placer mine.

At all sites, placer gold mining provided a series of cuts (25–500 m length) that presented frozen sediments, from which fossil nests and loess were collected at different depths. Some sites (QC, EC and SC; Fig 1) contain marker horizons in the form of the Dawson tephra (ca. 25.3 ka BP, [78]) and the Sheep Creek tephra (ca. 80 ka BP, [74, 79]).

Most fossil nests were completely frozen at the time of sampling (labeled ‘F’). Samples or portions of samples that had thawed were labeled ‘T’. In some cases, parts of a single nest were frozen, but had thawed recently where exposed by the mining cut. Frozen and thawed portions were analyzed separately. An average composition was reported, if no significant isotopic differences were observed between the ‘F’ and ‘T’ portions. All nests were kept frozen until prepared for analyses, at which time they were freeze-dried.

Distinctive macrofossils were collected from the freeze-dried nests, including seeds, leaves, stems, rodent bones, insects and hair. Plant macrofossils were identified to the closest possible taxonomic resolution by comparison with Zazula et al. [68, 75, 80]. Radiocarbon dates of plant tissues (leaf, stem) and/or bone samples from 10 nests plus two wood samples from the QC and IC sites were obtained (S1 Table) from the NSF-Accelerator Mass Spectrometry facility at the University of Arizona, Tucson, Arizona, USA.

Tahmasebi et al. [9] described collection and analysis of modern plants from east of Kluane Lake and the Whitehorse area of Yukon Territory (Fig 1). Modern ground squirrel bones were also collected from these areas (3 bones from Kluane Lake in 2013; 11 individuals from Erik Nielsen Whitehorse International Airport in 2014).

Microscopic examination of plant tissues was performed using a Leica S8APO-MDG41 dissecting microscope. Some samples were also mounted on an Al-stub, coated with Au-Pd alloy, and examined using a Hitachi S3400N scanning electron microscope operated at 25.0 kV.

### 2.2 Decomposition experiment

Above-ground portions of six modern plant species (*Poa glauca*, *Elymus trachycaulus*, *Artemisia frigida*, *Calamagrostis purpurascens*, *Festuca altaica*, *Elymus spicatus*) were collected from loess-fed grasslands east of Kluane Lake. These species were among the most common plants in eastern Beringia during the Late Pleistocene [68, 81–84]. Six wooden boxes were prepared and divided in half using a layer of polystyrene. The bottom of each box was covered with loess (2 cm) from site QC. Air-dried tissues of each plant species were then cut into ~2–3 cm-long pieces, and a layer (2 cm) placed on top of the loess, one species per box. The plants in half of each box were then covered with 2 cm of loess (‘buried condition’) to simulate underground nest conditions, while the other half of each box remained uncovered (‘not buried’ condition).

The boxes were placed outside in London, ON, Canada from October 21, 2013 to September 2, 2014. The plant tissues sampled at the start of the experiment (day 1), and then after 164, 253 and 317 days. Monthly mean temperature and total precipitation data for this period were obtained from Environment Canada for the weather station closest (~8 km) to the site of the decomposition experiment (S2 Table). The day-164 sampling (April 2, 2014) followed a very cold winter; samplings at days-253 and -317 occurred during summer (June 30 and September 2, 2014). To test for reproducibility, three aliquots of plant tissue were taken from different locations in each ‘not buried’ and each ‘buried’ portion of each box during each sampling. The samples were washed with distilled water (DW), dried at 90°C, ground and stored in glass vials prior to analysis.

### 2.3 Sample preparation

Visibly well-preserved fossil plant materials were sampled from each nest and soaked in DW three times, each time for 1–2 minutes, to disperse attached sediment. The samples were then washed with DW, dried overnight at 90°C, ground using a Wig-L-Bug^®^ (Crescent), and stored in small sealed glass vials while awaiting analysis. A similar preparation was used for modern plant samples [9].

Collagen was extracted from bone following Metcalfe et al. [85]. Bone bioapatite structural carbonate was assessed for *post-mortem* alteration using the Crystallinity Index (CI) and carbonate/phosphate ratio (C/P) obtained by Fourier Transform Infrared Spectroscopy (FTIR), following Webb et al. [86]. Precision was ± 0.11 for CI and ± 0.08 for C/P. Secondary carbonate was not detected in the FTIR spectra of any sample, and therefore no treatment for secondary carbonate removal was performed prior to isotopic analysis.

About 0.5–1 mg of crushed bone was reacted with ortho-phosphoric acid (H_3_PO_4_) under vacuum at 90°C for 25 minutes using a Micromass MultiPrep automated sampling device. The CO_2_ released was automatically transferred to a VG Optima isotope ratio mass spectrometer (IRMS), operated in dual-inlet mode, for measurement of *δ*^13^C, following the protocol of Metcalfe et al. [87] without any pretreatment for removing OM.

The preparation and analysis of loess for grain size, OM content, pH and mineralogy followed methods described by Tahmasebi et al. [9]. Carbonate removal from the loess was performed using acid fumigation [88].

### 2.4 OC and TN abundances and stable isotope analyses

Abundances of OC and total nitrogen (TN) in fossil plants and loess (after carbonate removal), and their carbon- and nitrogen-isotope compositions, were determined using an Elemental Analyzer (EA) (Costech Analytical Technologies, Valencia, CA, USA) coupled to a Thermo Scientific Delta^PLUS^ XL IRMS (Thermo Scientific, Bremen, Germany). The average C and N contents for the keratin standard was 47.74 ± 0.97 wt. % (n = 58) and 14.26 ± 0.44% (n = 113), respectively, which compare well to their expected values of 48.22 ± 1.07 wt. % and 14.85 ± 0.43 wt. %. The average N content for NIST 1547 was 2.72 ± 0.07 wt. % (n = 50), which compares well with its accepted value of 2.94 wt. %. Sample reproducibility for C was ± 0.43 wt. % (23 replicates) and for N was ± 0.02 wt. % (30 replicates).

All stable isotope results are presented using *δ*-notation [89], and related to VPDB for carbon and AIR for nitrogen using two-point calibrations. Plant and collagen C- and N-isotope compositions were calibrated using USGS40 and USGS41 [90]. Using this calibration, the average *δ*^13^C and *δ*^15^N obtained for an internal keratin standard were ‒24.04 ± 0.07 ‰ (n = 75) and +6.42 ± 0.12 ‰ (n = 113), respectively, which compare well with their accepted values of –24.05 ± 0.15 ‰ and +6.36 ± 0.22 ‰, respectively. The average *δ*^13^C obtained for IAEA-CH-6 was ‒10.50 ± 0.09 ‰ (n = 26), which compares well with its accepted value of ‒10.45 ± 0.03 ‰. Sample reproducibility was ± 0.15 ‰ for *δ*^13^C (26 replicates) and ± 0.08 ‰ for *δ*^15^N (32 replicates).

Structural carbonate C-isotope compositions (*δ*^13^C_Sc_) were calibrated to VPDB using NBS 19 and LSVEC [91]. Using this calibration, the average *δ*^13^C obtained for internal calcite standards WS-1 and Suprapur were +0.69 ± 0.11 ‰ (n = 3) and ‒35.78 ± 0.01 ‰ (n = 2), respectively, which compare well with their accepted values of +0.76 ‰ and ‒35.55 ‰, respectively.

### 2.5 Statistical analysis

Changes in *δ*^13^C and *δ*^15^N of plant detritus during the decomposition experiment were tested using repeated measures ANOVA (general linear model) and applying the Greenhouse-Geisser correction. When the time effect on *δ*^13^C and *δ*^15^N was significant, the Bonferroni *post hoc* test was used to perform Pairwise Comparisons to determine at what interval the significant difference occurred. Possible correlations between decomposed plant *δ*^15^N and C/N or C content were assessed using Pearson’s rank correlation coefficient (R). All statistical analyses were performed in SPSS 20.

## 3 Results

### 3.1 Loess

Loess *δ*^15^N ranges from +1.3 ‰ in QC-4 to +4.8 ‰ in IC-9 and QC-5; variation in *δ*^13^C is smaller (‒26.1 to ‒25.4 ‰) (Table 2). Physical and chemical properties of the loess are given in Table A in S1 Text.

**Table 2:** Isotopic composition of loess TN and OC.

Sample ID	*δ*^15^N (‰, AIR)	*δ*^13^C (‰, VPDB)
**QC-2**	+4.6	‒25.8
**QC-3**	+3.4	‒25.7
**QC-4**	+1.3	‒26.1
**QC-5**	+4.8	‒25.6
**QC-6**	+4.7	‒25.4
**QC-7**	+4.3	‒25.4
**QC-8**	+4.5	‒25.4
**IC-9**	+4.8	‒25.4

### 3.2 Plants

#### 3.2.1 Fossil plant botanical composition

**Fig 2:**
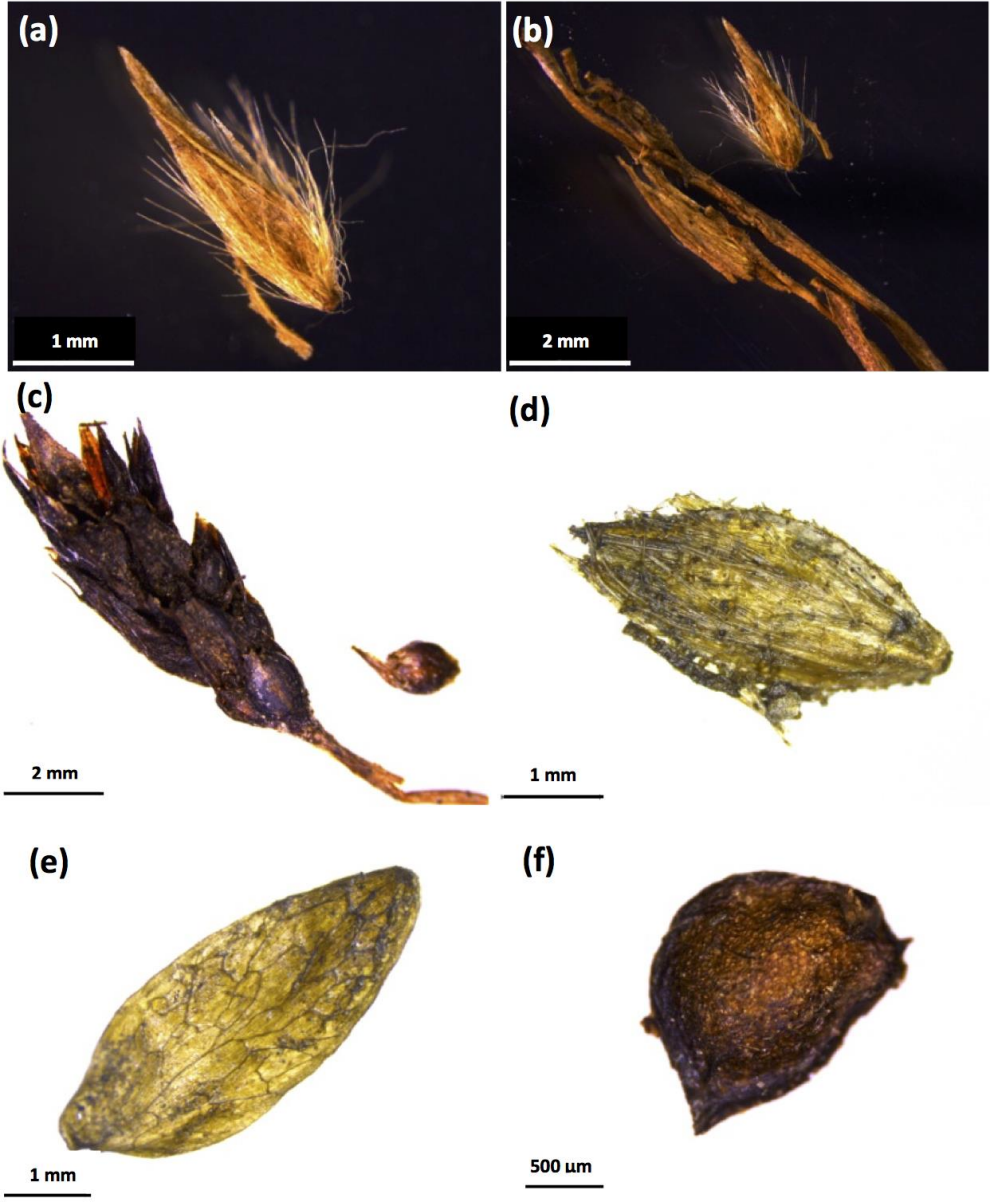
Typical plant macrofossils recovered from fossil nests. (a) *Deschampsia cespitose* floret; (b) *Deschampsia cespitosa* floret with stem; (c) *Carex albonigra* floret and seed; (d) *Alopecurus* sp. floret; (e) *Draba* sp. Silique; (f) *Ranunculus eschscholtzii-sulphureus* type achene.

**Fig 3:**
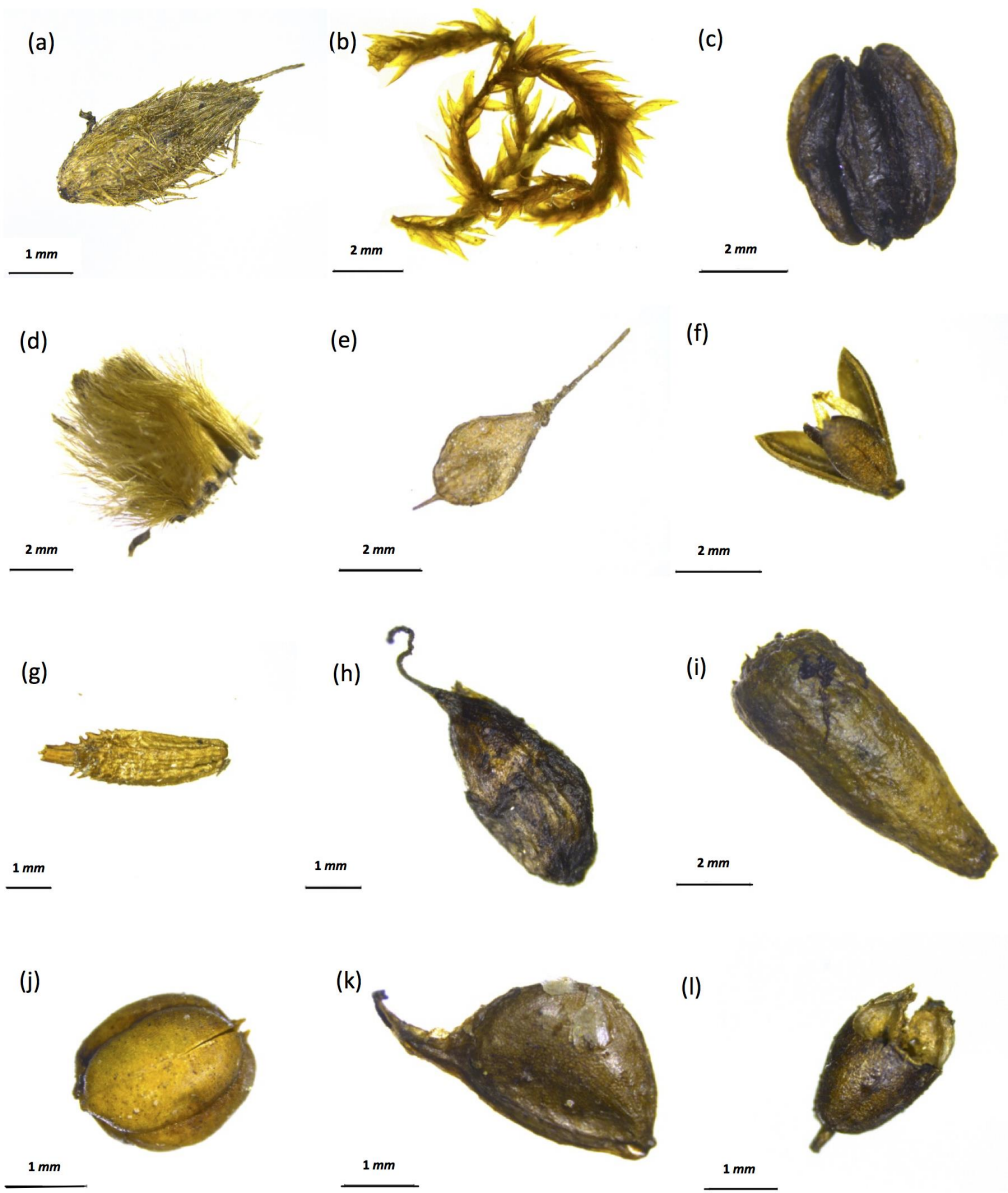
Typical plant macrofossils recovered from fossil nests. (a) *Carex* floret; (b) *Campylium stellatum* stem with leaves; (c) *Conioselinum cnidii folium* carpel; (d) *Asteracea* achenese, pappus; (e) *Lepidium densiflorum*; (f) *Phlox hoodii* capsule; (g) *Taraxacum ceratophorum* achene; (h) *Pedicularis* sp. achene; (i) *Silene* cf. *taymirensis* capsule with seeds inside; (j) *Polemonium* capsule; (k) *Ranunculuc pensylvanicus-macounii* type achene; (l) *Plantago* cf. *canescens* capsule.

Plant macrofossils recovered from the nests are dominated by grass florets (*Alopecurus* sp., *Deschampsia caespitose* and *Carex* spp.) and the dried fruits of forbs (*Taraxacum* sp., *Draba* sp., *Ranunculus* sp., *Lepidium densiflorum* and *Plantago* cf. *canescens*) (Figs 2 and 3). These observations are consistent with previous descriptions of Late Pleistocene Beringia as a grass- and forb-dominated ecosystem [68, 83, 84, 92].

#### 3.2.2 Plant C and N contents, stable isotope compositions and dating

The *δ*^13^C of the fossil plant samples ranges from ‒27.7 to ‒24.1 ‰ (avg. ‒26.1 ‰); values of *δ*^15^N exhibit much larger variation (+1.0 to +13.6 ‰; avg. +5.5 ‰) (Table 3). The N and C contents of all samples range from 1.0 to 2.8 wt. % and 21.2 to 39.5 wt. %, respectively, and have an average atomic C/N of 24.0 ± 6.0 (Table 3). Dates obtained for 10 selected samples from fossil nests (S1 Table) range from >49.9ka BP to 16.58ka BP. An age range for each sampling site was estimated based upon the oldest and youngest radiocarbon date obtained at each site (Table 1).

**Table 3:** C and N contents and isotopic compositions for fossil plants.

Nest ID	*δ*^13^C	*δ*^15^N	C	N	Atomic C/N
	(‰)		(wt. %)		
QC-1-T*	‒26.6	+4.6	29.5	1.7	20.8
**QC-2-F**	‒24.1	+2.1	31.4	1.4	25.8
**QC-3-F**	‒26.2	+1.1	28.1	1.6	20.4
**QC-3-T**	‒26.1	+2.4	33.5	1.8	21.8
**QC-4-T**	‒26.1	+1.9	31.4	1.6	22.4
**QC-5-F**	‒26.1	+1.8	32.2	2.0	19.0
**QC-5-T**	‒27.7	+2.1	32.1	2.0	18.5
**QC-7-F**	‒26.2	+2.6	38.7	1.1	39.6
**QC-8-F**	‒25.6	+7.5	36.0	1.8	22.9
**QC-9-F**	‒25.6	+9.3	32.3	2.8	13.4
**QC-GZ-2-3**	‒26.1	+6.6	39.5	1.3	36.2
**QC-GZ-5-9**	‒26.4	+3.9	37.9	1.4	32.3
**QC-GZ-1**	‒25.7	+3.3	29.8	1.3	26.9
**IC-1-F**	‒26.7	+10.3	31.5	1.9	19.8
**IC-1-T**	‒25.6	+9.0	39.2	2.2	20.4
**IC-2-F**	‒26.6	+6.2	30.8	1.6	22.2
**IC-3-F**	‒26.4	+10.6	36.2	2.1	20.2
**IC-4-F**	‒26.9	+1.2	28.6	1.6	21.3
**IC-5**-**F**	‒26.5	+9.2	35.8	1.8	23.7
**IC-6-F**	‒27.6	+10.0	30.2	1.4	26.1
**IC-7-F**	‒25.7	+4.6	38.1	1.4	31.1
**IC-7-T**	‒25.3	+4.3	25.4	1.6	18.9
**IC-8-F**	‒25.9	+6.3	36.0	1.6	25.6
**IC-8-T**	‒26.8	+8.5	33.5	1.7	22.7
**IC-9-F**	‒27.0	+2.4	34.9	1.8	22.6
**IC-9-T**	‒26.3	+1.9	36.1	1.7	24.2
**IC-10-F**	‒26.0	+2.7	33.6	1.7	23.8
**IC-11-F**	‒26.4	+4.2	32.8	1.4	28.3
**IC-12-F**	‒26.3	+5.2	34.0	1.7	23.7
**IC-13-T**	‒25.2	+3.4	36.5	1.8	24.1
**IC-14-F**	‒26.2	+4.4	36.0	1.7	24.2
**IC-14-T**	‒26.1	+3.6	30.8	1.6	23.1
**IC-15-T**	‒25.9	+2.1	25.8	1.5	19.8
**IC-19-T**	‒26.2	+6.3	35.8	1.7	24.6
**LB-GZ-1**	‒26.3	+7.1	31.4	2.0	18.5
**GC-GZ-3**	‒25.7	+7.4	21.2	2.1	11.8
**EC-GZ-6**	‒25.6	+7.5	36.6	1.7	24.8
**EC-GZ-2**	‒26.8	+4.8	29.0	1.9	18.1
**SC-GZ-8**	‒24.9	+5.8	29.3	1.7	20.7
**SC-GZ-7**	‒26.5	+1.0	38.7	1.0	44.3
**SC-GZ-4**	‒26.2	+5.2	30.1	1.3	27.8
**SC-GZ-2**	‒24.7	+8.5	38.9	1.7	27.3
**SC-GZ-10**	‒26.0	+13.6	33.4	1.5	26.3

*T: Thawed at the time of sampling; F: Frozen at the time of sampling.

Average *δ*^13^C and *δ*^15^N of all modern plant parts reported by Tahmasebi et al. [9] vary as follows: (i) Kluane Lake, *δ*^13^C = ‒27.1 ± 1.2 ‰ (n = 207), and *δ*^15^N = ‒0.1 ± 2.2 ‰ (n = 207); Whitehorse area, *δ*^13^C = ‒27.8 ± 1.2 ‰ (n = 115), and *δ*^15^N = +0.2 ± 2.2 ‰ (n = 115).

### 3.3 Bone isotopic compositions

Fossil bones recovered from the fossil nests consisted mostly of Arctic ground squirrels (IC-3, IC-19, IC-9-2, IC-14) and lemmings (GZ-1, GZ-3-1, IC-9-1, QC-4). Two bone samples, which differed in size and morphology, were selected from each of nests GZ-3 and IC-9. Specimen GZ-3-2 most probably belongs to a megaherbivore (unknown species), and was not included in isotopic averages for the rodents.

Bone collagen N contents (N >10 wt. %), atomic C/N (3.2 to 3.6) and extraction yields (>2%) (Table 4) indicate excellent collagen preservation [93]. Fossil rodent bone *δ*^13^C_Col_ and *δ*^15^N_Col_ range from ‒21.9 to ‒19.9 ‰ (avg. ‒21.3 ‰), and +3.9 to +5.6 ‰ (avg. +4.6 ‰), respectively (Table 4). Modern bone *δ*^13^C_Col_ and *δ*^15^N_Col_ are lower, ranging from ‒24.2 to ‒21.4 ‰ (avg. ‒23.2 ‰), and +1.1 to +3.2 ‰ (avg. +2.1 ‰), respectively (Table 4). The average *Δ*^13^C_Col-Bulk plant_ and *Δ*^15^N_Col-Bulk plant_ for the modern bone collagen samples are +4.7 ‰ and +1.9 ‰, respectively, based on the average *δ*^13^C and *δ*^15^N obtained for the modern plants from each site (Whitehorse and Kluane Lake; see Tahmesabi et al. [9]).

**Table 4:** Bone *δ*^13^C_Col_, *δ*^13^C_Sc_, *δ*^15^N_Col_, C_Col_ and N_Col_.

ID	*δ*^13^C_Col_	*δ*^13^C_Sc_	*Δ*^13^C_Sc-Col_	*δ*^15^N_Col_	C	N	C/N	Collagen
	(‰ VPDB)			(‰, AIR)	(wt. %)		(atomic)	Yield (%)
**Fossil bone**								
**QC-4**	‒21.4	‒13.1	+8.3	+4.8	37.6	13.2	3.3	8.5
**IC-3**	‒21.4	‒14.5	+6.9	+5.1	32.8	11.5	3.3	4.5
**IC-9-1**	‒21.7	‒14.3	+7.4	+4.0	42.4	14.7	3.4	5.1
**IC-9-2**	‒21.5	‒14.0	+7.6	+4.2	39.6	13.8	3.4	6.5
**IC-14**	‒21.2	‒12.5	+8.7	+3.9	28.7	9.3*	3.6	2.3
**IC-19**	‒21.2	‒13.2	+8.0	+4.6	43.8	15.9	3.2	15.3
**LB-GZ-1**	‒21.9	‒14.5	+7.4	+4.5	43.6	15.2	3.4	8.6
**GC-GZ-3-1**	‒19.9	‒12.0	+7.9	+5.6	42.4	15.0	3.3	9.1
**GC-GZ-3-2**	‒19.5	-	-	+5.3	32.5	11.4	3.3	4.4
**Modern**								
**M-1-female**^**1**^	‒23.8	‒18.3	+5.4	+1.3	48.3	16.8	3.4	15.4
**M-2-male**^**1**^	‒24.2	‒18.7	+5.6	+2.8	41.7	14.7	3.3	16.5
**M-3-male**^**1**^	‒23.8	‒17.7	+6.1	+2.8	47.9	16.7	3.4	15.7
**M-5-male**^**1**^	‒23.9	‒17.6	+6.2	+2.1	41.0	14.6	3.3	17.2
**M-6-male**^**1**^	‒22.7	‒16.9	+5.9	+2.3	42.2	14.7	3.4	16.4
**M-7-male**^**1**^	‒23.5	‒17.6	+6.0	+1.1	46.4	15.9	3.4	14.6
**M-8-female**^**1**^	‒23.7	‒18.3	+5.4	+1.9	47.5	16.5	3.4	15.3
**M-9-male**^**1**^	‒23.4	‒17.7	+5.7	+3.2	40.5	14.4	3.3	14.3
**M-10-male**^**1**^	‒23.3	‒18.3	+5.0	+2.4	41.1	14.6	3.3	16.7
**M-11-male**^**1**^	‒23.5	‒17.9	+5.6	+1.8	39.9	14.1	3.3	16.9
**M-12-male**^**1**^	‒23.9	‒18.9	+5.0	+2.0	42.2	15.0	3.3	15.3
**M-14**^**2**^	‒21.7	‒9.6	+11.6	+1.8	43.9	15.6	3.3	7.0
**M-15**^**2**^	‒22.7	‒8.9	+13.8	+1.8	41.8	14.3	3.4	12.9
**M-16**^**2**^	‒21.4	‒8.8	+12.6	+1.9	36.2	12.8	3.3	8.9

^1^ Sampling site: Whitehorse.

^2^ Sampling site: East of Kluane Lake.

* Only 0.06 mg of the sample was available for analysis because of the small size of the bone fragment.

Mean FTIR-CI values of modern and fossil bone bioapatite are 2.5 ± 0.1 (n = 14) and 2.6 ± 0.2 (n = 8), respectively (S3 Table), typical of unaltered material [94]. The mean C/P of both modern and fossil bones is 0.7 ± 0.1 (S3 Table), which is only slightly higher than well-preserved bone (~0.5) [95]. No secondary calcite or francolite was observed in the FTIR spectra. Hence, the *δ*^13^C_Sc_ results are considered to reflect *in vivo* conditions. The *δ*^13^C_Sc_ of fossil bone ranges from ‒14.5 to ‒12.0 ‰ (avg. ‒13.5 ‰), and the *Δ*^13^C_Sc-Col_ varies from +6.9 to +8.7 ‰ (avg. +7.8 ‰) (Table 4). For the modern bone samples, the *δ*^13^C_Sc_ varies considerably between the two study areas, ranging from ‒9.6 to ‒8.8 ‰ (avg. ‒9.1 ‰) at Kluane Lake *vs*. ‒18.9 to ‒16.9 ‰ (avg. ‒18.0 ‰) at Whitehorse (Table 4). The *Δ*^13^C_Sc-Col_ at Kluane Lake varies from 11.6 to 13.8 ‰ (avg. +12.7 ‰) *vs*. +5.0 to +6.2 ‰ (avg. +5.6 ‰) at Whitehorse.

## 3.4 Modern plant decomposition

### 3.4.1 Isotopic data

**Fig 4:**
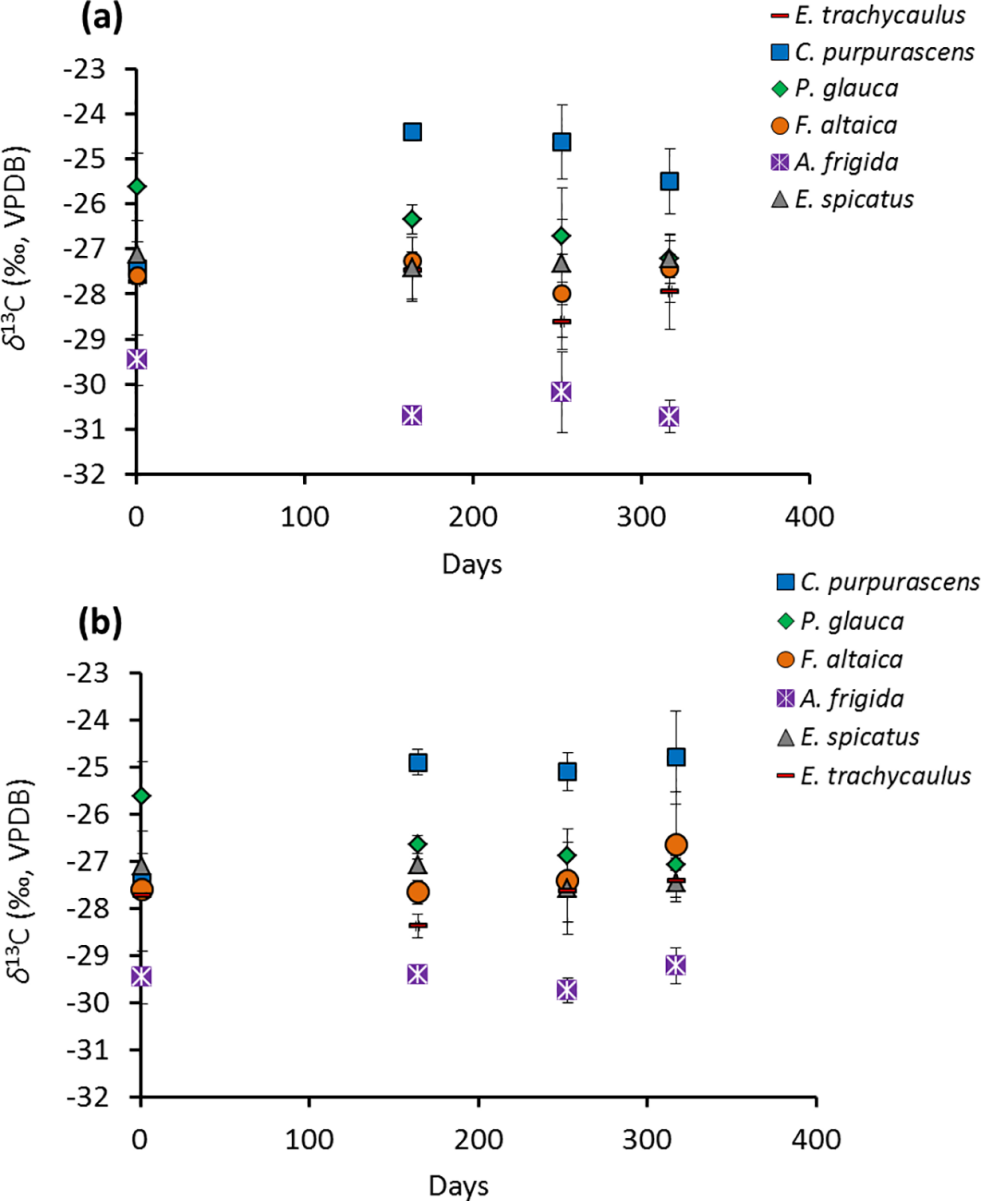
Values of *δ*^13^C_litter_ *vs*. time during decomposition: (a) ‘buried’ and (b) ‘not buried’.

**Fig 5:**
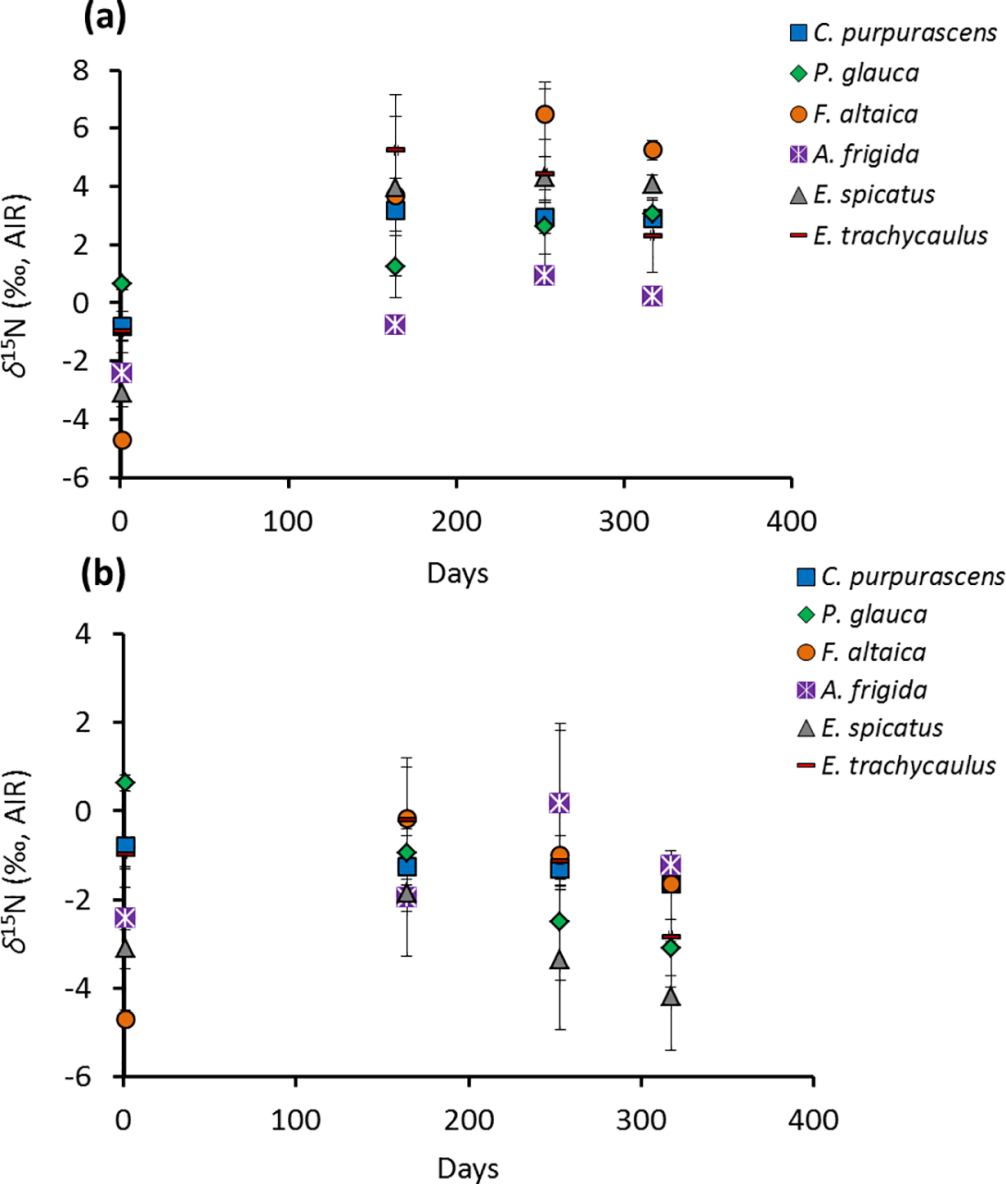
Values of *δ*^15^N_litter_ *vs*. time during decomposition: (a) ‘buried’ and (b) ‘not buried’.

Figs 4A and 4B and 5A and 5B illustrate the change in average plant litter *δ*^13^C (*δ*^13^C_litter_) and *δ*^15^N (*δ*^15^N_litter_), respectively, over 317 days, for ‘buried’ and ‘not buried’ samples, respectively (See Table A in S1 File for all data). Table 5 summarizes the repeated measure ANOVA results for the time effect on *δ*^13^C_litter_ and *δ*^15^N_litter_ during this experiment. For samples showing a significant time effect on their isotopic compositions, the Benferroni *post hoc* test results are summarized in Supporting Information (Table A for *δ*^13^C and Table B for *δ*^15^N in S2 File).

**Table 5:** Repeated measure ANOVA test results for time effects on *δ*^13^C_litter_ and *δ*^15^N_litter_.

Plant ID	Treatments	*δ*^13^C (‰, VPDB)		*δ*^15^N (‰, AIR)	
		*p*	F(X,Y)	*p*	F(X,Y)
***E*. *trachycaulus***	**B**	0.210	F(1.69,3.38) = 2.54	**0.042**	**F(1.53,3.07) = 10.94**
	**NB**	0.241	F(1.22,0.52) = 2.48	0.256	F(1.29,2.57) = 2.26
***C*. *purpurascens***	**B**	**0.021**	**F(1.53,3.06) = 17.92**	**0.008**	**F(1.67,3.34) = 29.95**
	**NB**	0.052	F(1.10,1.93) = 14.94	0.795	F(1.32,2.64) = 0.14
***P*. *glauca***	**B**	0.148	F(1.08,2.16) = 4.95	**0.034**	**F(1.98,3.96) = 8.96**
	**NB**	0.131	F(1.25,2.50) = 5.04	**0.004**	**F(1.87,3.74) = 34.91**
***F*. *altaica***	**B**	0.351	F(1.20,2.40) = 1.44	**0.012**	**F(1.98,3.96) = 16.46**
	**NB**	0.316	F(1.00,2.01) = 1.76	0.327	F(1.00,2.01) = 1.65
***A*. *frigida***	**B**	0.230	F(1.37,2.73) = 2.52	**0.050**	**F(1.03,2.07) = 17.53**
	**NB**	0.480	F(1.97,3.95) = 0.89	0.227	F(1.43,2.86) = 2.51
***E*. *spicatus***	**B**	0.842	F(1.12,2.23) = 0.07	0.079	F(1.06,2.11) = 10.38
	**NB**	0.589	F(1.06,2.12) = 0.43	0.371	F(1.44,2.88) = 1.30

B: Buried

NB: Not Buried

Values in boldface are statistically significant (*p* ≤ 0.05).

*C*. *purpurascens* is the only species showing a significant shift in *δ*^13^C (from ‒27.5 ‰ to ‒25.5 ‰ for the ‘buried’ sample), particularly in the first 164 days (Table A in S2 File). A much larger change is observed in *δ*^15^N_litter_ for the buried samples, ranging from an increase of ~2.4 ‰ for *P*. *glauca* to an increase of ~10.0 ‰ for *F*. *altaica* (Fig 5A). This change is statistically significant for all species except *E*. *spicatus* (Table 5). For all samples, most of the ^15^N-enrichment occurred in the first 164 days. For ‘not buried’ samples, only *P*. *glauca* shows a significant change in *δ*^15^N, which is characterized by a progressive decrease in ^15^N (Table 5; Table B in S2 File).

#### 3.4.2 C and N contents, atomic C/N, and SEM

**Fig 6:**
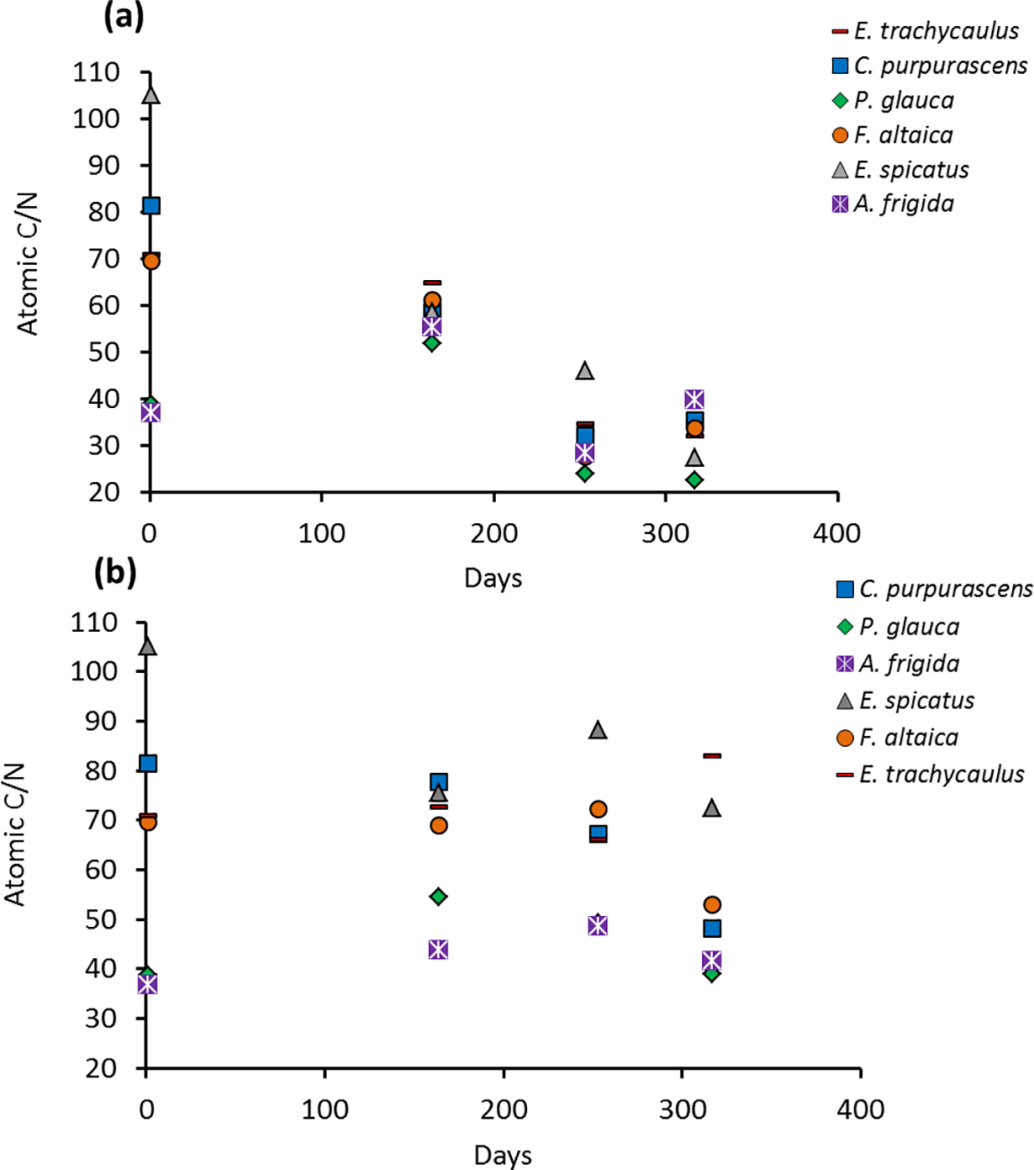
Atomic C/N *vs*. time during decomposition: (a) ‘buried’ and (b) ‘not buried’.

**Fig 7:**
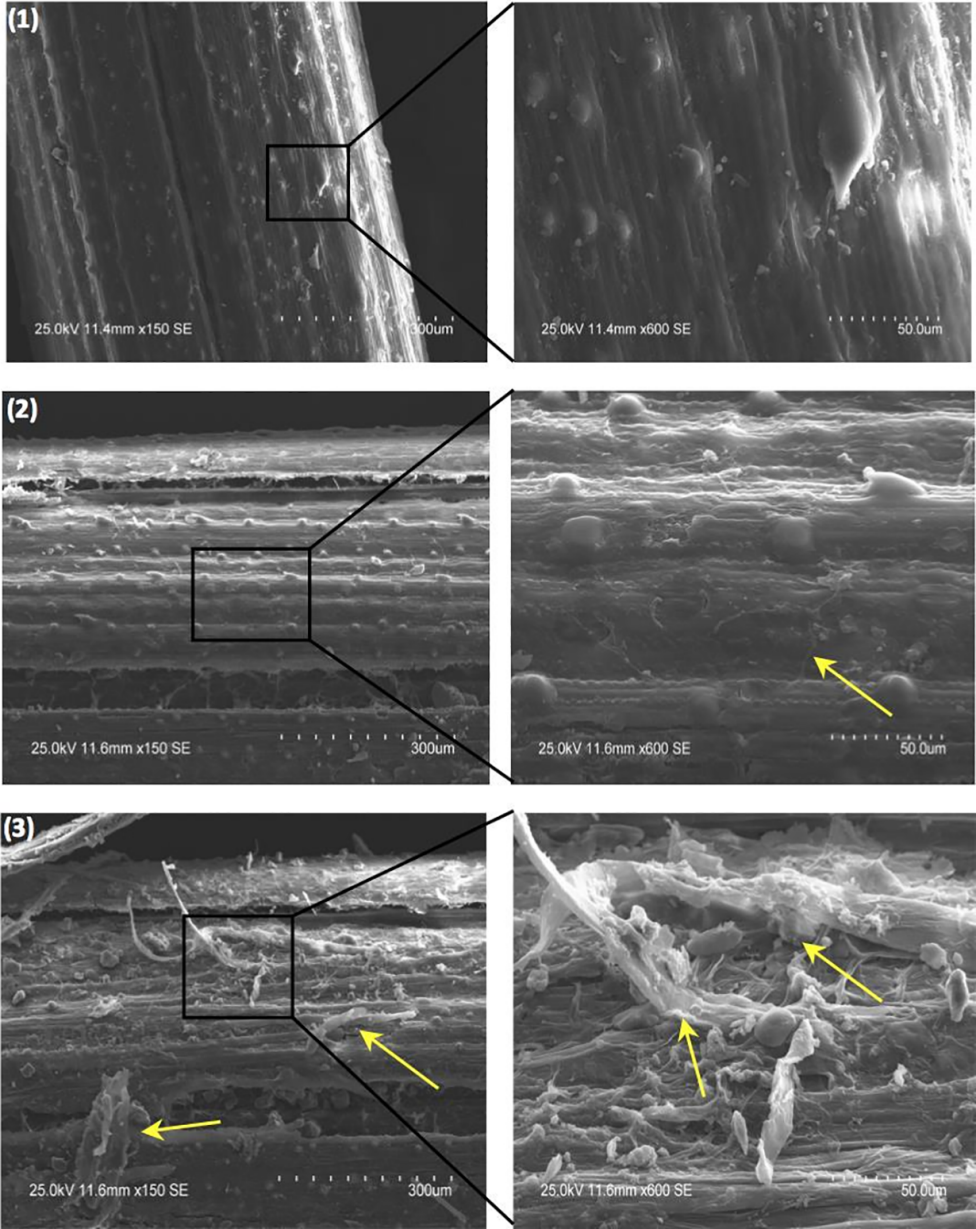
SEM images of *F*. *altaica*: (1) before decomposition, (2) after 317 days of decomposition without a covering of loess (‘not buried’), and (3) after 317 days of decomposition under a thin covering of loess (‘buried’). (Yellow arrows point to fungal hypha).

All data are listed in Supporting Information Tables A, B and C in S1 File. Atomic C/N of the starting materials ranges from 36.9 to 105.2 (Fig 6A and 6B), with *P*. *glauca* and *A*. *frigida* having the lowest ratio (<50), mainly because of their higher N contents relative to other species. During the burial experiment, the atomic C/N of all species decreased, except for *A*. *frigida* (Fig 6A). *P*. *glauca* and *E*. *spicatus* show the smallest (16.3) and largest (77.7) changes, respectively. For ‘not buried’ samples, there is no consistent pattern of change in atomic C/N (Fig 6B). ‘Buried’ plant detritus has higher visible abundances of fungal hyphae than ‘not buried’ equivalents (Fig 7).

## 4 Discussion

### 4.1 Modern plant decomposition experiment

In general, the *δ*^13^C_litter_ does not show a clear time-dependent pattern during decomposition, either for ‘buried’ or ‘not buried’ samples. *C*. *purpurascens* is the only species showing a significant change (^13^C-enrichment), which occurs in the first 164 days of the ‘buried’ treatment.

The large increase in *δ*^15^N_litter_ (2.5–10 ‰; Fig 5A) observed here for ‘buried’ *vs*. ‘not buried’ samples (Fig 5B), coupled with the decrease in C/N (16–78; Fig 6A) and the abundance of fungal hyphae on the surfaces of the former (Fig 7), suggests a link with microbially mediated decomposition of the litter. Greater decomposition of ‘buried’ *vs*. ‘not buried’ plant detritus can be explained by a buffering role for soil in balancing pH, water and oxygen availability, all of which would favour decomposers [96]. In particular, soil water content undergoes greater fluctuation at the soil surface because of the former’s direct exposure to wind, light and rain.

The appearance of earthworms in the ‘buried’ plant detritus at sampling days 253 and 317 is a potential complication. Earthworms are rare in Yukon Territory at present, and their importance in Beringia over the time span considered here is unknown. Relative to other detritivores, earthworms can accelerate OM decomposition rates [97–99]. Earthworms are also known to lower SOM C/N via digestion driven by their gut microbial community [100]. The abundant fungal hyphae observed in our experiment, however, suggest that bacterial communities related to earthworm arrival had not become dominant relative to fungal decomposers, and hence our analogy to an earthworm-free system remains valid. We note also that most change in C/N of the ‘buried’ plant detritus occurred prior to earthworm arrival.

Most ^15^N-enrichment of ‘buried’ samples occurred during the first 164 days (Fig 5A), when the most labile plant material would have been in greatest abundance. The samples also received the most precipitation during this time and were insulated from the coldest surface temperatures by loess and snow cover, which are both conditions that favour decomposition.

Dried *P*. *glauca* and *A*. *frigida*, which had much lower initial C/N than other species examined, showed much less change in C/N during decomposition, and in ‘buried’ treatment they showed only minor variation in *δ*^13^C_litter_ or *δ*^15^N_litter_. ‘Not buried’ samples behaved similarly, except for a significant lowering of *δ*^15^N_litter_ for *P*. *glauca*. Why *P*. *glauca* and *A*. *frigida* did not show greater ^15^N enrichment during early decomposition may be related to litter quality [101], which can affect microbially mediated decomposition. *Artemisia* contains essential oils, anti-herbivory alkaloids and anti-fungal secondary metabolites, which inhibit bacterial and fungal processes [102, 103].

**Fig 8:**
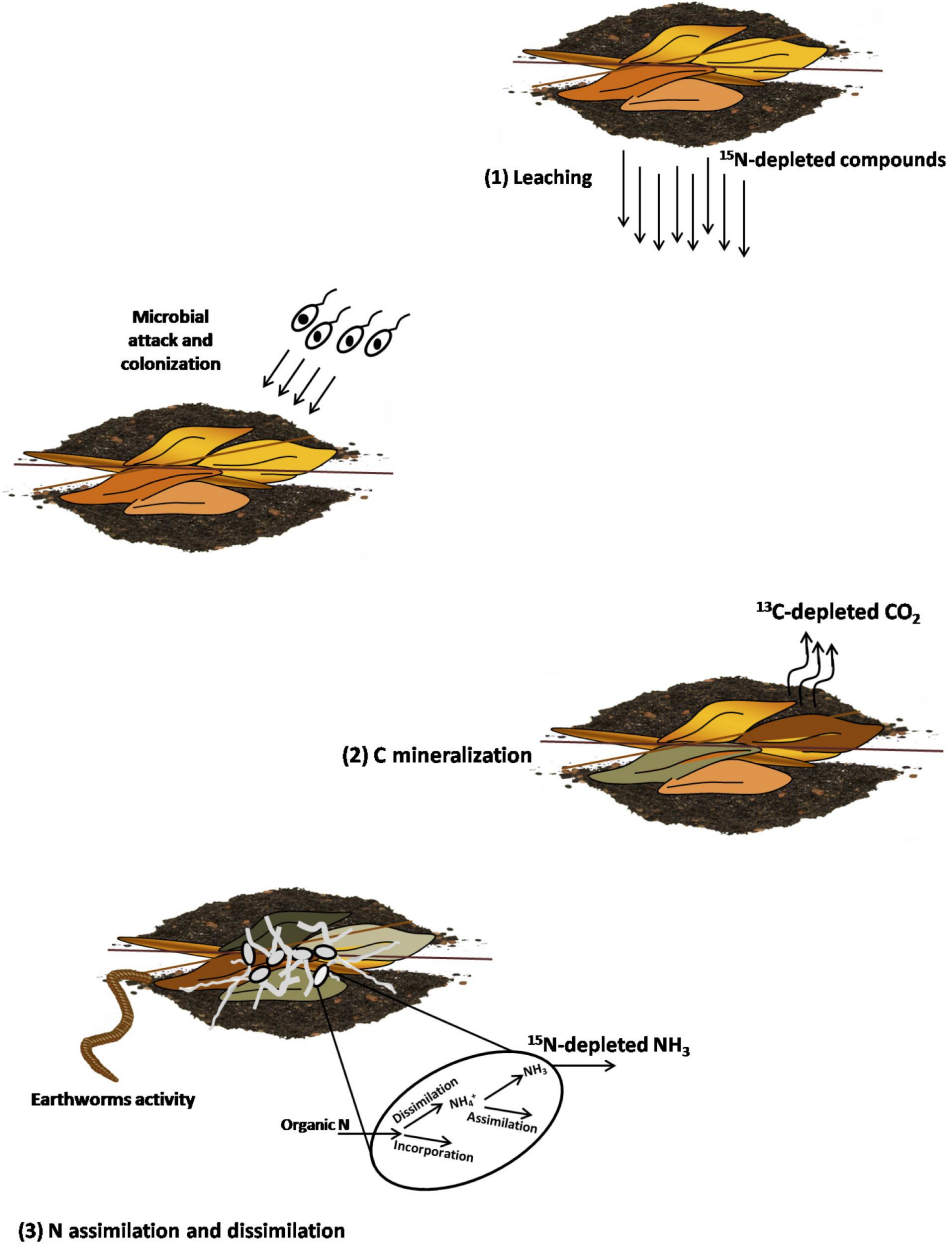
**Processes causing** ^**13**^**C- and** ^**15**^**N-enrichment of ‘buried’ decomposing plants:** (1) leaching of ^15^N-depleted compounds [104, 105]; (2) C mineralization and release of ^13^C-depleted CO_2_, and (3) microbial N metabolism (release of ^15^N-depleted NH_3_; enrichment of microbes in ^15^N [106]).

**Fig 9:**
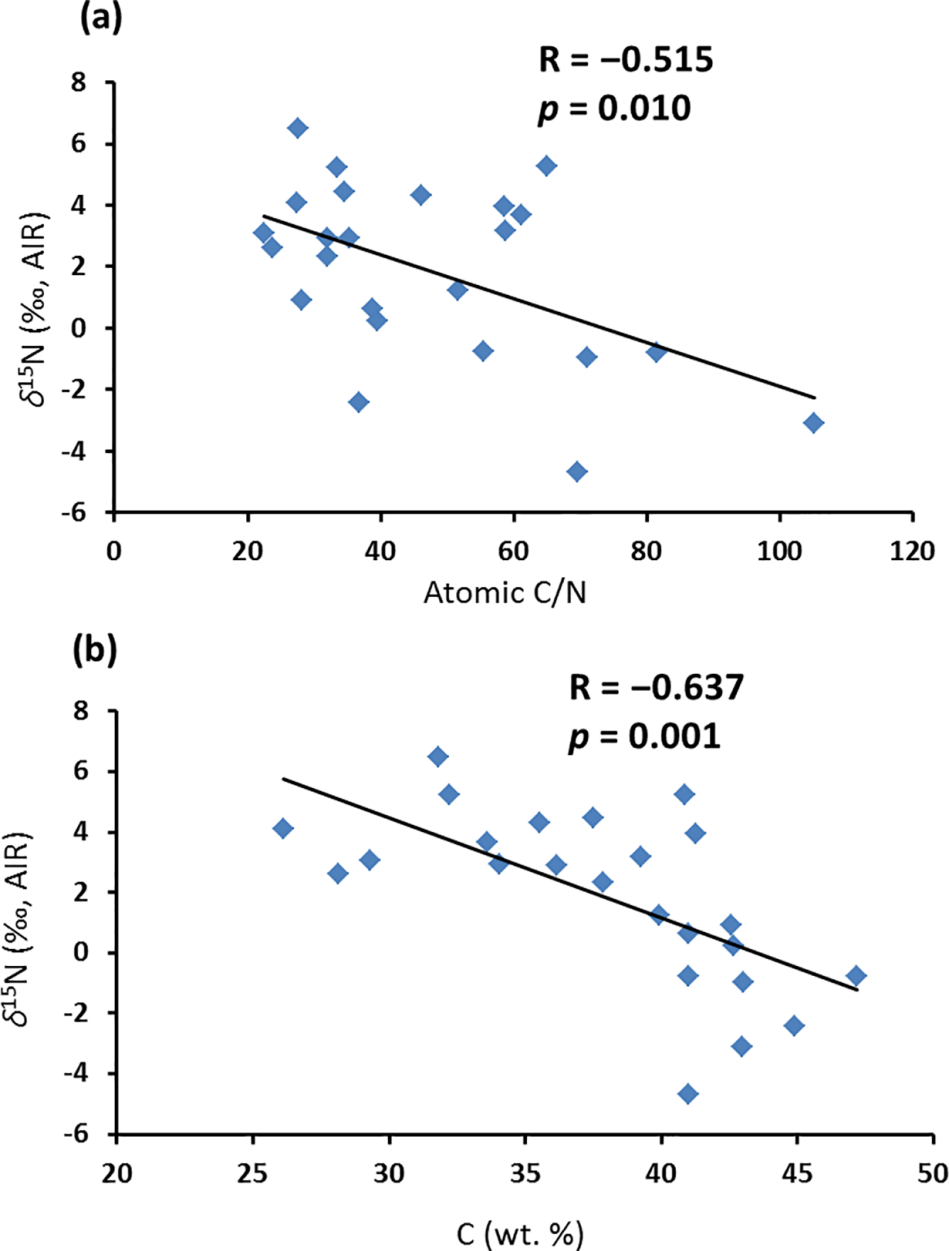
Bivariate plot of (a) *δ*^15^N *vs*. atomic C/N, and (b) *δ*^15^N *vs*. C (wt. %) of decomposed plant debris.

Fig 8 presents a model for ^13^C-enrichment of ‘buried’ *C*. *purpurascens* and ^15^N-enrichment of most species examined. Leaching early in decomposition [104] enriches the system in ^15^N by removal of low-^15^N compounds [105]. Microbial respiration releases ^13^C-depleted CO_2_, leaving microbial products enriched in ^13^C and causing a decrease in atomic C/N. The negative correlation between *δ*^15^N and both atomic C/N and C (wt. %) for all six species over 317 days (Fig 9) is similar to that between microbial ^15^N-enrichment and soil-soluble C/N reported by Dijkstra et al. [106]. They suggested that early stages of plant decomposition are characterized by microbial assimilation of N, while N dissimilation is more prevalent in later stages. Once labile C-compounds are consumed during early decomposition, microbes then consume more N-rich compounds, dissimilating portions of organic N into NH_4_^+^. After some NH_4_^+^ is assimilated, the remainder is released as NH_3_ (Fig 8). N-isotope fractionation during these processes leads to ^15^N-enrichment of microbes and release of ^15^N-depleted NH_3_ [31]. While the putative effect of earthworm gut contents on *δ*^15^N_litter_ is unknown, their nutrient-rich casts most probably contribute N, adding further complexity to the simple system described above. Based on the pattern of change in C/N, which can be explained mostly by C loss (Fig 8), significant change in bulk N content of the decomposed plant tissues plus microbial biomass is unlikely. A few patterns remain unexplained, such as the decrease in *δ*^15^N of ‘not buried’ *P*. *glauca* as decomposition progressed. This experiment may not have reached steady state by the end of 317 days.

We suggest that plant tissues underwent similar isotopic alteration following incorporation into Pleistocene arctic ground squirrel nests. Therefore, consideration of decomposition isotopic effect in interpreting isotopic data from fossil plants is necessary. Decomposition of senesced plants at the ground surface, however, likely had little effect on plant isotopic compositions.

### 4.2 Loess

The loess samples are dominated by silt (Table A in S1 Text), characteristic of other Klondike loess and associated paleosols [73, 74], and their mineralogy (quartz, feldspar, calcite) (Table B in S1 Text) is typical of Quaternary loess worldwide [107]. The OM content (5–20 wt. %) and high abundance of fine and very fine roots in all loess samples suggest a base-rich environment during the Late Pleistocene and that below-ground plant parts were a major source of OM [74]. As also noted by Sanborn et al. [74], the presence of ground squirrel nests and the lack of peat layers (OC >17 wt. %) indicate well-drained conditions in the past. The average loess *δ*^13^C of ‒25.6 ‰ is typical of pre-Industrial Revolution C_3_ vegetation. Most loess samples have a high and tight range of *δ*^15^N (+3.4 to +4.8 ‰). The one outlier (QC-4; *δ*^15^N = +1.3 ‰) represents a dark layer between two lighter loess layers, and has the highest OM (20.4 wt. %) and OC (9.5 wt. %) contents of the samples analyzed (Table A in S1 Text). These features may indicate a very cold period during which decomposition was limited.

### 4.3 Plant macrofossils

The *δ*^13^C of plant macrofossils (‒27.7 to ‒24.1 ‰) from the Pleistocene ground squirrel nests indicates C_3_ vegetation and is consistent (after Suess effect correction) with earlier results for modern [9, 37] and Late Pleistocene fossil plants from eastern Beringia [92, 108]. The positive *δ*^15^N (majority >+2 ‰) of the arctic ground squirrel nests is higher on average than reported for modern plants [9] and Late Pleistocene plant macrofossils (avg. ‒2.8 ‰) [92] from eastern Beringia. The N contents of the squirrel nest plants lie in the range known for modern subarctic plants [9, 37]. The C contents are lower than reported for modern plants [9, 37], but similar to Late Pleistocene fossil plants from eastern Beringia [37]. These results point to possible changes in the original isotopic and elemental compositions of the fossil plants comprising the arctic ground squirrel nests.

### 4.4 Fossil and modern bone collagen and bioapatite structural carbonate

The average *δ*^13^C_Col_ of fossil bones (‒21.3 ± 0.7 ‰) is similar to the range reported by Bocherens et al. [109] for four post-LGM ground squirrels (‒21.0 to ‒20.3 ‰) from Switzerland, which date to 14–12 ka BP. Assuming *Δ*^13^C_Col-diet_ of +3 to +4 ‰ [110] and *Δ*^13^C_Sc-diet_ of +9.9 ‰ [49], the average *δ*^13^C_Col_ (‒21.3 ± 0.7 ‰) and *δ*^13^C_Sc_ (‒13.5 ± 1.0 ‰) of these fossil bones suggest an entirely C_3_ diet. This result is consistent with previous paleovegetation reconstructions for eastern Beringia [37, 92].

The average *δ*^13^C_Col_ of the modern ground squirrel bones (‒23.2 ± 0.8 ‰) also compares well with results for the fossil bones (after Suess effect correction (~+2.2 ‰)). The average *δ*^13^C_Sc_ for these samples, however, differs between localities. At Whitehorse, the average *δ*^13^C_Sc_ (‒18.0 ± 0.6 ‰) indicates an entirely C_3_ diet, whereas the average *δ*^13^C_Sc_ (‒9.1 ± 0.4 ‰) at Kluane Lake suggests a mixed diet of C_3_ plants and more ^13^C-rich vegetation. The Kluane Lake *δ*^13^C_Sc_ can be explained if protein (represented by collagen) was derived mainly from the C_3_ portion of the diet, whereas whole diet (represented by structural carbonate), which includes carbohydrates and lipids, came from a C_4_ source or ^13^C-rich macrophytes. The high *Δ*^13^C_Sc-Col_ of Kluane Lake ground squirrels (+12.7 ± 1.1 ‰) is similar to that of captive rats raised on a mixed diet of C_3_-proteins and C_4_-carbohydrates and lipids [49, 50]. A significant presence of C_4_ plants in subarctic regions is not expected [111], although C_4_ plants (e.g. *Muhlenbergia richardsonis*) are known from the Kluane Lake area (Consortia of Pacific Northwest Herbaria: B. A. Bennett Herbarium). Wooller et al. [37] also reported a few C_4_ grasses from Alaska and Yukon with *δ*^13^C ranging from ‒14 to ‒12 ‰. Consumption of high-^13^C macrophytes is also possible given the study area’s proximity to Kluane Lake at its junction with the Slims River. There, very shallow water provides an ephemeral home for submergent macrophytes, which are exposed on river/delta flats during seasonal dry conditions. Consumption of high-^13^C macrophytes or C_4_ plants that likely have low protein content (as reported for *M*. *richardsonis* by Dittberner and Olsen [112]), could increase consumer’s *δ*^13^C_Sc_, without significantly changing *δ*^13^C_Col_.

The *δ*^15^N_Col_ of the fossil bones (+3.9 to +5.6 ‰) is much higher than reported by Bocherens et al. [109] (+1.7 to +2.5 ‰) for the post-LGM ground squirrels from Switzerland. Much of this greater enrichment in ^15^N is unlikely to be related to significant consumption at higher trophic levels, given the largely herbivorous nature of arctic ground squirrels, a condition also implied by the Beringian samples’ *Δ*^13^C_Sc-Col_ (+6.9 to +8.7 ‰) [45]. That said, minor consumption of insects, small invertebrates, and other carrion cannot be ruled out. The implications of this difference in N-isotope composition are discussed next.

### 4.5 Late Pleistocene Beringia and modern comparisons

There is no exact modern analogue for Late Pleistocene Beringia [4, 27]. Some portions of west-central Yukon, however, such as the eastern shoreline of Kluane Lake, may be broadly comparable in climatic conditions (windy, arid, low temperature), sediment and soils (continuous loess deposition, high pH, high OM) and vegetation (an *Artemisia*-*Festuca* grassland) [73, 81] [9, 68, 83, 84]. Local factors including elevation, topography, strength of loess deposition, drainage, aspect, slope and water content, which are superimposed upon the regional patterns, have determined the ecological mosaics at both modern Kluane Lake and ancient eastern Beringia [80, 81]. The south-central Whitehorse valley, located farther to the east from Kluane Lake, has similar continental, cold and dry climate and grassland vegetation.

### 4.6 Modern and ancient C and N isotope baselines

**Fig 10:**
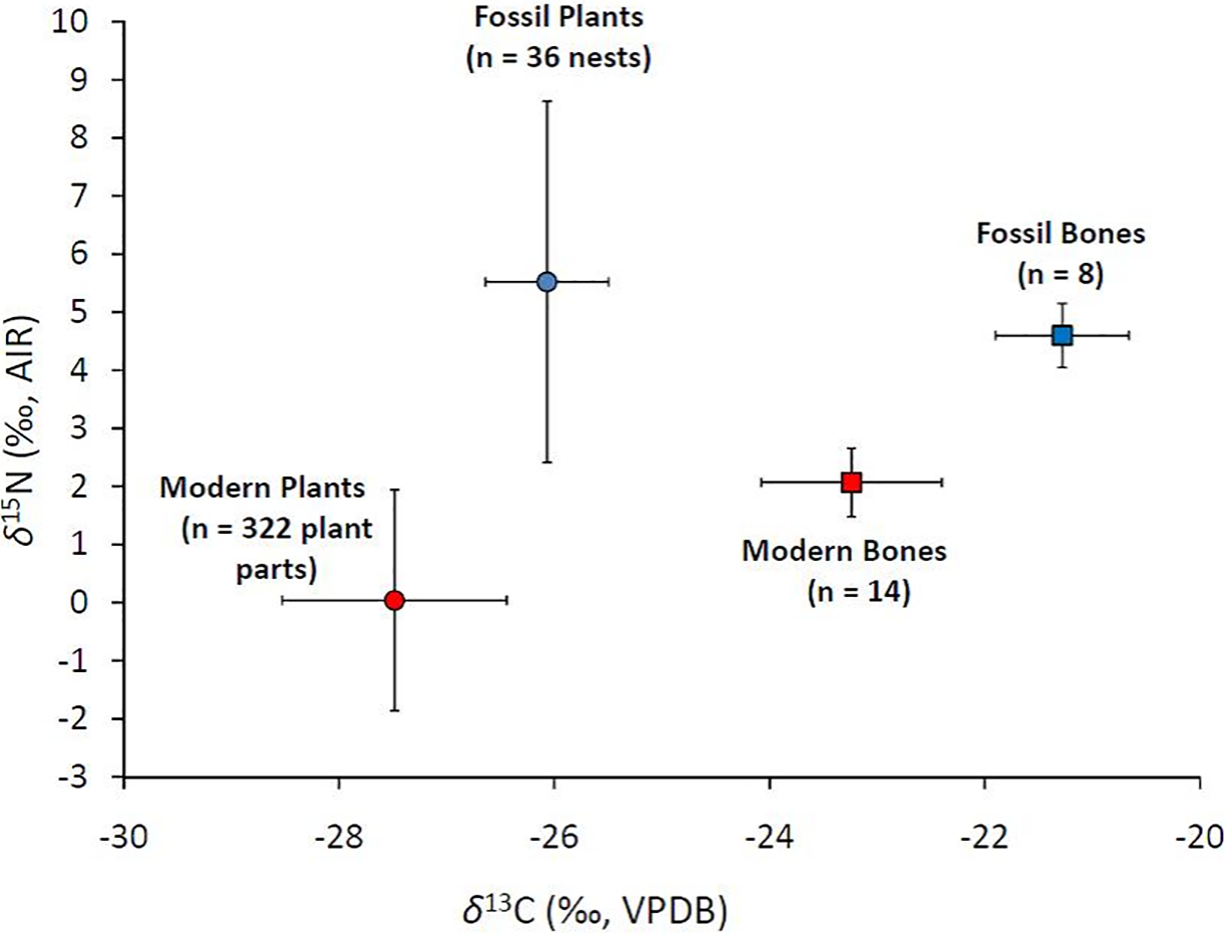
Average *δ*^15^N *vs*. *δ*^13^C of fossil and modern samples.

The higher average *δ*^13^C and *δ*^15^N of fossil plants and bone collagen relative to their modern equivalents (Fig 10) could point to a change in N- and C-isotope food web baselines in this ecosystem between the Late Pleistocene and present time. The lower average *δ*^13^C of modern plants (by ~1.4 ‰) and modern arctic ground squirrel bone collagen (by ~2.0 ‰) relative to their ancient counterparts can be largely explained by the ~2.2 ‰ decrease in *δ*^13^C_atm_ arising from the Suess effect. The difference between modern and ancient plant *δ*^13^C, however, is ~0.8 ‰ smaller than predicted from the change in *δ*^13^C_atm_. One possible explanation is that low-level microbial processes further modified the *δ*^13^C of ancient vegetation during its residence in permafrost.

The fossil plants and fossil bone collagen also have higher average *δ*^15^N than their modern counterparts (Fig 10). The difference for plants (~5.5 ‰), however, is twice as large as for bones (+2.5 ‰). Part of the higher plant *δ*^15^N could indicate a different rate of N dynamics in Beringia during the Late Pleistocene than at present time. The much larger size of this difference between fossil and modern plants compared to fossil and modern bone collagen, however, suggests that fossil plant *δ*^15^N was also affected by other factors.

**Fig 11:**
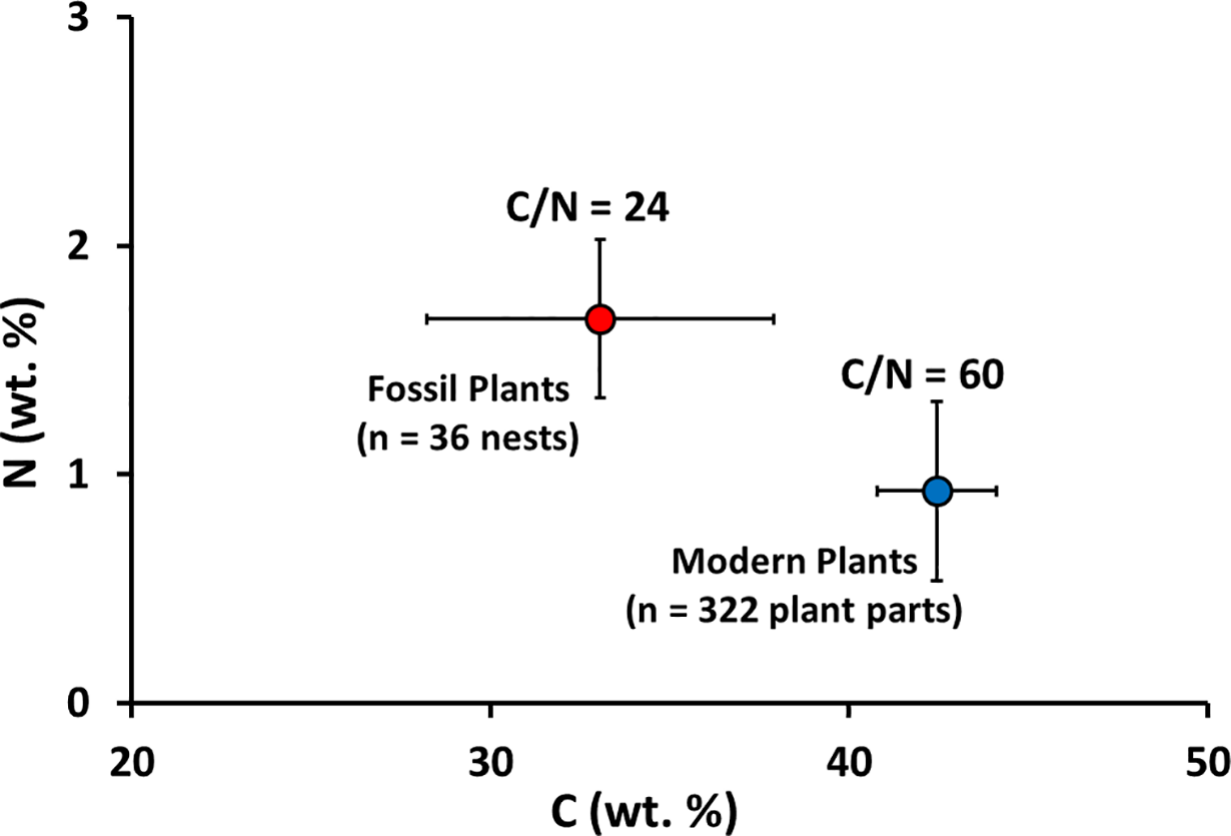
Average N *vs*. C contents of fossil and modern plants.

**Fig 12:**
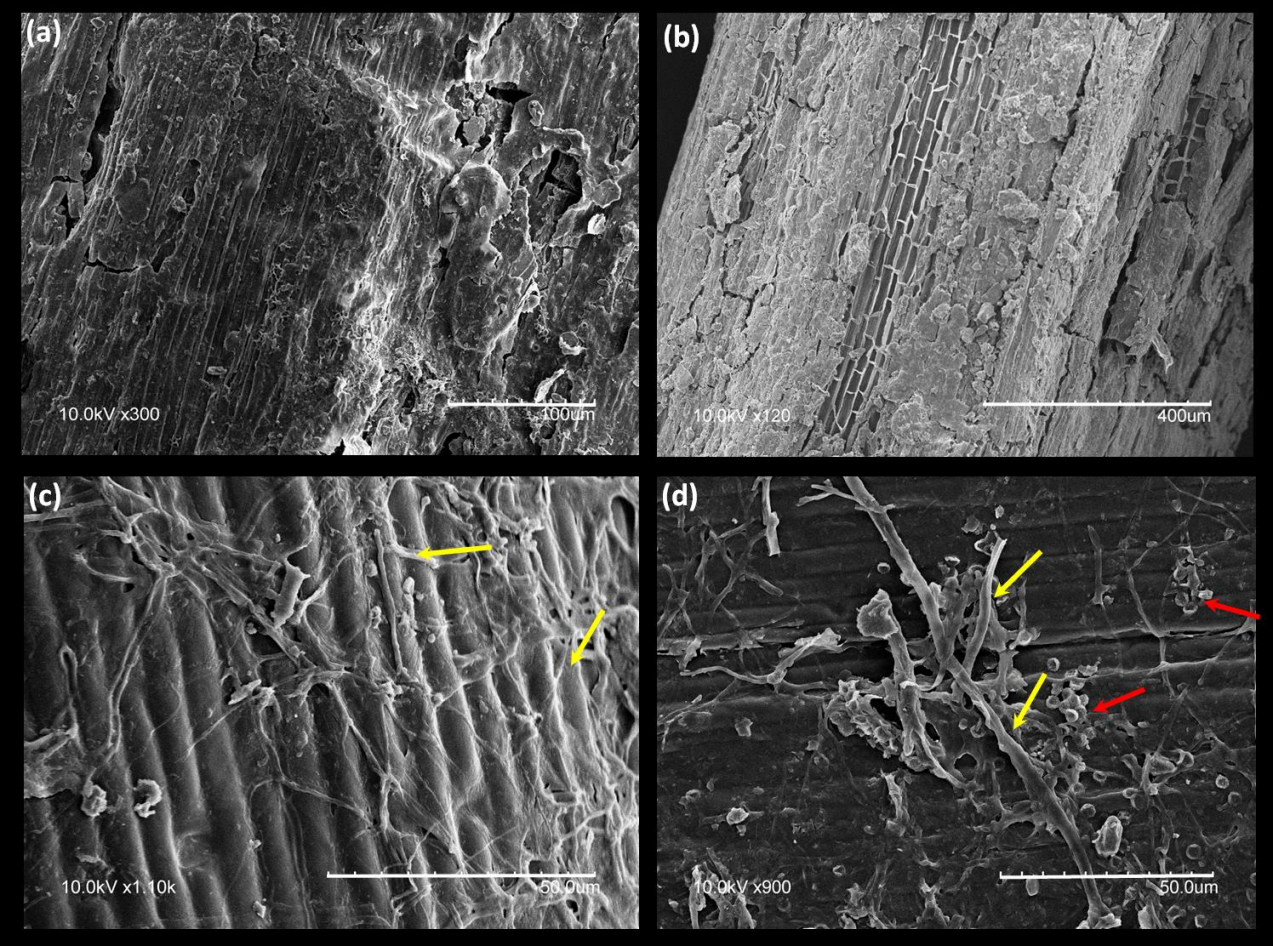
**SEM images of plant macrofossils:** (a, b) typical decomposed surface of fossil stems, and (c, d) fungal hypha (yellow arrows) and bacteria cells (red arrows) on fossil stem surfaces.

Three lines of evidence suggest that microbially mediated decomposition modified the original N-isotope composition of fossil plants. First, the fossil plants have higher N contents, lower C contents, and significantly lower atomic C/N than the modern plants (Fig 11), consistent with the results of the decomposition experiment. Second, SEM images of plant macrofossils indicate plant tissue alteration and establishment of fungal hyphae and bacteria (Fig 12). Third, if ground squirrels/lemmings ate similar plant material to that stored in their nests, then the *δ*-values for their diet should match those measured for these plants, assuming modern values for *Δ*^13^C_Col-bulk plant_ (4.7 ‰) and *Δ*^15^N_Col-bulk plant_ (1.9 ‰).

**Fig 13:**
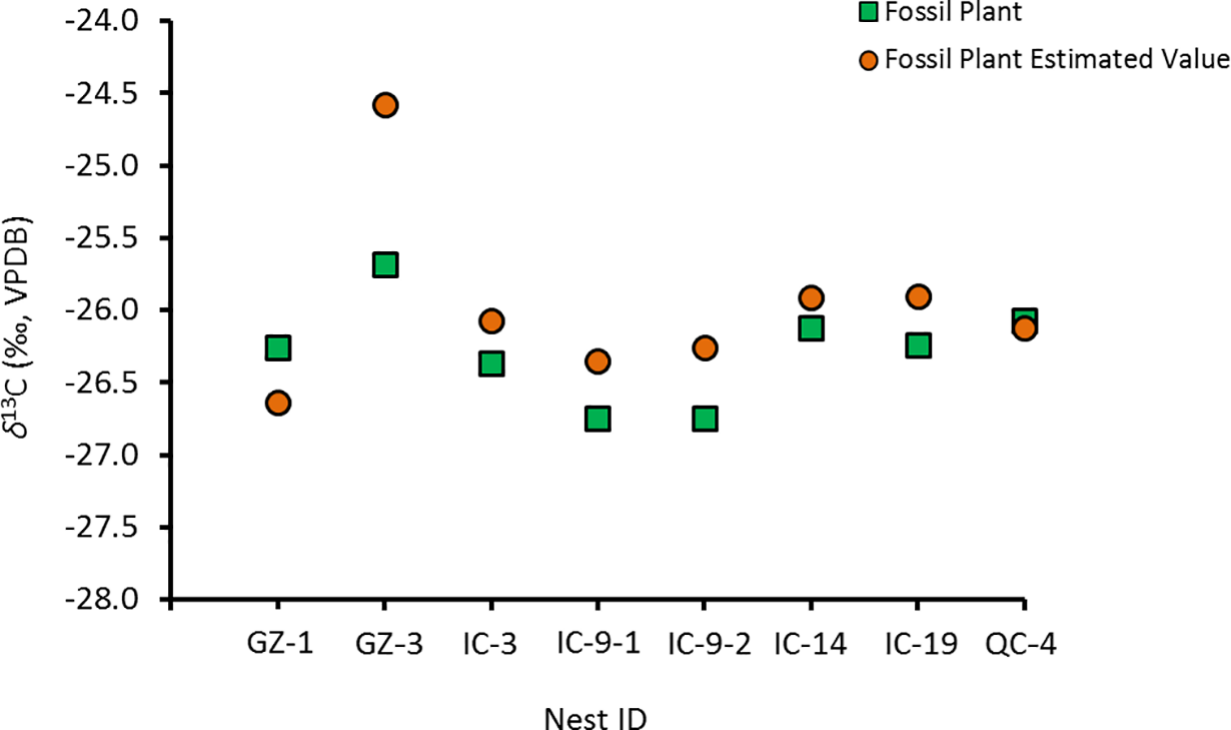
Measured *δ*^13^C *vs*. predicted *δ*^13^C for fossil plants using fossil bone *δ*^13^C and modern *Δ*^13^C_Col-Bulk plant_ = 4.7 ‰.

**Fig 14:**
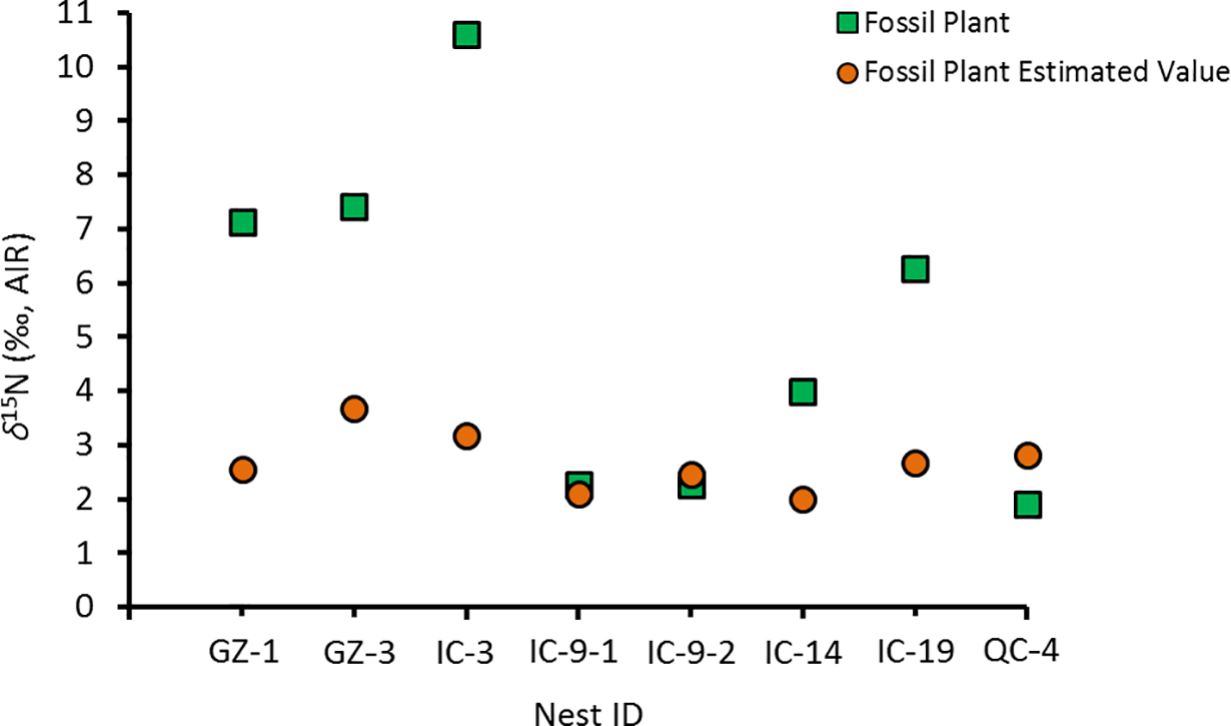
Measured *δ*^15^N *vs*. predicted *δ*^15^N for fossil plants using fossil bone *δ*^15^N and modern *Δ*^15^N_Col-Bulk plant_ = 1.9 ‰.

For *δ*^13^C, the match is good, except for one sample (GZ-3) (Fig 13). For *δ*^15^N, only two nests (IC-9 and QC-4) have measured *δ*^15^N close to predicted values (Fig 14).

**Fig 15:**
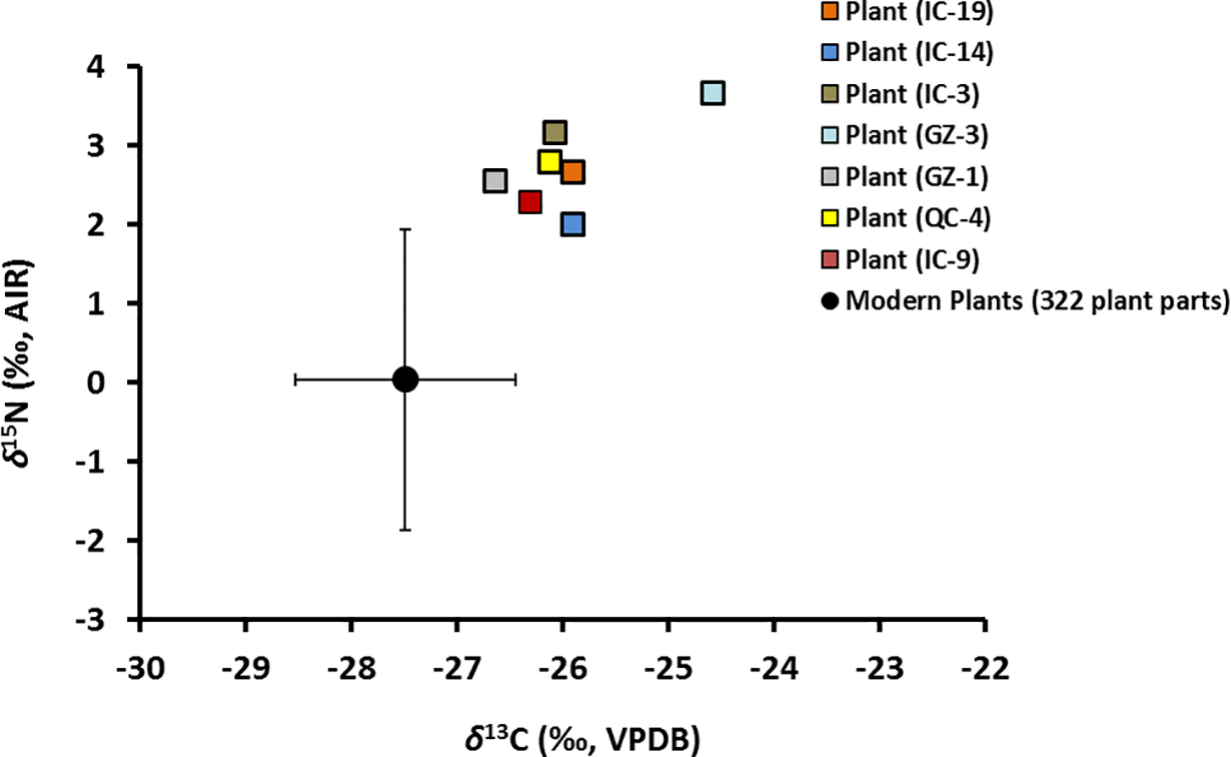
Values of *δ*^15^N *vs*. *δ*^13^C of plant macrofossils after correction for the isotopic effects of decomposition (squares). The mean (filled circle) and range (whiskers) of *δ*^15^N and *δ*^13^C for modern plants from the region [9] are shown in black.

The fossil plant isotopic compositions were corrected for the effects of decomposition using the isotopic discrimination factors measured for modern bone collagen and plants (*Δ*^13^C_Col-bulk plant_ and *Δ*^15^N_Col-bulk plant_). These discrimination factors were applied to the C- and N-isotope compositions of fossil bones collected from the same nests. Following this correction, the calculated average *δ*^13^C of the fossil plants is still higher than that of the modern plants (~1.5 ‰) (Fig 15), which can be explained for the most part by the Suess effect. The calculated average *δ*^15^N of the fossil plants is ~2.8 ‰ higher than modern plants from the region (Fig 15). This difference, we suggest, indicates that the food web *δ*^15^N baseline was higher in the Late Pleistocene than at present, which is consistent with a more open N cycle.

While plant tissues and bone collagen of the Late Pleistocene samples have higher *δ*^15^N than their modern equivalents, a similar pattern is not observed for loess. The average *δ*^15^N (~+4.1 ± 1.2 ‰; n = 8) of the Late Pleistocene loess is similar to that of modern equivalents (~+4.9 ± 1.6 ‰; n = 14) sampled from various depths at Kluane Lake [9]. Our data are insufficient to explain this similarity. Perhaps bulk sediment *δ*^15^N is not representative of bioavailable N [31]. Also, it is unknown if the ancient loess samples analyzed in this study had experienced weak pedogenesis. Position within a soil profile can exert a strong control on soil *δ*^15^N (e.g. [31, 33, 113]).

Another intriguing consideration is the role of biological soil crusts (BSC) in the N cycle in such grassland ecosystems [114, 115]. The abundance of lichen-dominated BSC in the Kluane Lake area, for example, has been suggested as the most likely source of new N addition to the soil [114]. BSE likely plays a very significant role in regulating the productivity of these grasslands at present. At Kluane Lake, for example, the N content of BSC is ~8-times higher than the underlying mineral soil [114]. As also reported by Marsh et al. [114], the BSC *δ*^15^N in the Kluane Lake area is significantly lower than the underlying mineral soil: +3.1±1.5 ‰ for the mineral soil versus +2.2±1.7 ‰ for bulk BSC in one set measurements, and –0.2±0.6 ‰ for upper BSC and +1.6±0.8 ‰ for lower BSC in a second set of measurements. The lower BSC *δ*^15^N signals atmospheric N fixation as the prevailing process responsible for this N. What remains to be learned in future research, however, is whether BSC was important during Beringian times.

The significant and continuous environmental changes that accompanied disappearance of large mammals at the terminal Pleistocene throughout the Holocene, together with rising atmospheric *p*CO_2_ that followed, could have led to a gradual shift in N dynamics in eastern Beringia. More isotopic data from fossil samples with a continuous chronology spanning Late Pleistocene to modern time in this region is required to further verify the results and interpretations presented here, and clarify the main triggers of the proposed N-isotope shift.

## 5 Conclusions

Comparison of *δ*^15^N and *δ*^13^C of fossil terrestrial plants and rodent bone collagen from Late Pleistocene, eastern Beringian localities in Yukon Territory with modern equivalents indicate a change in the C- and N-isotope food web baseline. The plant *δ*^13^C change is explained mostly by the Suess effect on modern samples. Higher *δ*^15^N of the plant macrofossils relative to modern equivalents reflect: (i) microbially mediated decomposition of fossil plants, and (ii) a different N dynamic in the Late Pleistocene than at present time in these parts of Yukon Territory.

Eastern Beringia was an important end-member of the now-vanished Mammoth Steppe. Increasingly, changes in the size, position and extent of overlap among the isotopic niches of the assemblage of Mammoth Steppe megaherbivores, as defined by *δ*^13^C_Col_ and *δ*^15^N_Col_, are the tools of choice for inferring ecological changes over time in this now vanished megacontinental biome. Such analyses are then used to infer the climatic, anthropogenic or other processes that led to the Mammoth Steppe’s fragmentation and ultimate collapse–with attendant lessons for ecosystem destabilization during the current period of climate warming. Recognizing and then defining food web baseline shifts in *δ*^15^N and *δ*^13^C are a fundamental prerequisite to any such explanations of collagen stable isotopic data for Late Pleistocene megaherbivores from this region.

## Supporting information

S1 Table Radiocarbon dates for selected materials recovered from fossil nests. (DOCX)

S2 Table Weather data for London, ON, Canada (decomposition experiment). (DOCX)

S3 Table FTIRCI and C/P for fossil and modern bone bioapatite. (DOCX)

S1 Fig **Asimplified model for the “openness” of the N cycle in ecosystems with high (a) and low (b) N availability (from Tahmasebi et al. [9])**.
(**a**): (a) N mineralization: Conversion of organic N to NH_4_^+^ (ε = 0–5 ‰); (b) Microbial assimilation: incorporation of NH_4_^+^ into microbial biomass (ε = 14–20 ‰); (c) NH_3_ volatilization: conversion of NH_4_^+^_(aq)_ to NH_3(g)_ (ε = 40–60 ‰); (d) Nitrification: conversion of NH_4_^+^ to NO_3_^-^ (ε = 15–35 ‰); (e) Plant uptake and assimilation of NH_4_^+^ (ε = 9–18 ‰); (f) Plant uptake and assimilation of NO_3_^-^ (ε = 0–19 ‰); (g) NO_3_^-^ leaching (ε = 0–1 ‰); (h) Denitrification: conversion of NO_3_^-^ to N_2_O, N_2_ and NO_2_ (ε = 28–33 ‰).(**b**): (a) N mineralization: Conversion of organic N to NH_4_^+^ (ε = 0–5 ‰); (b) Microbial assimilation: incorporation of NH_4_^+^ into microbial biomass (ε = 14–20 ‰); (c) Nitrification: conversion of NH_4_^+^ to NO_3_^-^ (ε = 15–35 ‰); (d) Plant uptake and assimilation of NH_4_^+^ (ε = 9–18 ‰); (e) Plant uptake and assimilation of NO_3_^-^ (ε = 0–19 ‰). Values of ε are from Robinson [29] and Houlton and Bai [105]).
(TIF)

S1 Text Loess chemical and physical properties. **Table A:** Loess characteristics.
**Table B:** Loess mineralogy based on XRD.
(DOCX)

S1 File **Table A:** *δ*^13^C and *δ*^15^N for replicate analyses from the decomposition experiment.
**Table B:** C and N contents for replicate analyses from the decomposition experiment.
**Table C:** Atomic C/N of plant detritus during decomposition.
(DOCX)

S2 File **Table A:** Benferroni *post hoc* test for differences in average *δ*^13^C_litter_ between Day 1 and Days 164, 253 and 317 for samples showing significant time effect.
**Table B:** Benferroni *post hoc* test for differences in average *δ*^15^N_litter_ between Day 1 and Days 164, 253 and 317 for samples showing significant time effect.
(DOCX)
