# Supplementary material for: Nitrogen isotopes suggest a change in nitrogen dynamics between the Late Pleistocene and modern time in Yukon, Canada
Source: PLoS One. 2018 Feb 15;13(2):e0192713. doi: 10.1371/journal.pone.0192713 (PMC5813965; doi:10.1371/journal.pone.0192713)
Supplement: S1 Table — (DOCX) [file pone.0192713.s001.docx]

## S1 Table: Radiocarbon dates for selected materials recovered from fossil nests.

| **AA Lab#** | **Nest ID/site ID** | **Material** | **Radiocarbon age (yr BP)** |
| --- | --- | --- | --- |
| AA105552 | QC-4 | Bone fragment | >49,900 |
| AA105549 | IC-1 | Plant tissues | 24,800±180 |
| AA105548 | IC-3 | Plant tissue | 22,940± 140 |
| AA105550 | IC-14 | Plant tissue | >41,200 |
| AA105551 | IC-9 | Plant tissue | 26,930± 230 |
| AA105545 | IC-19 | Bone fragment | 22,520± 240 |
| AA105547 | LB-GZ-1 | Bone fragment | >40,300 |
| AA105546 | GC-GZ-3 | Bone fragment | 16,580± 120 |
| AA105553 | EC-GZ-2 | Plant tissue | 26,530± 220 |
| AA105554 | SC-GZ-10 | Plant tissue | 21,180± 110 |
| AA105555 | QC | Wood | >49,900 |
| AA105556 | IC | Wood | 32,320± 450 |

Sample ID from NSF-AMS Laboratory, University of Arizona, Tucson, Arizona, USA.
