# Supplementary material for: Nitrogen isotopes suggest a change in nitrogen dynamics between the Late Pleistocene and modern time in Yukon, Canada
Source: PLoS One. 2018 Feb 15;13(2):e0192713. doi: 10.1371/journal.pone.0192713 (PMC5813965; doi:10.1371/journal.pone.0192713)
Supplement: S2 Table — (DOCX) [file pone.0192713.s002.docx]

**S2 Table:** Weather data for London, ON, Canada (decomposition experiment).

| **Month** | **MAT (ᵒC)^1^** | **MTP (mm)^2^** |
| --- | --- | --- |
| **2013^3^** | | |
| **October** | +11.5 | 155.3 |
| **November** | +1.6 | 51.4 |
| **December** | ‒4.5 | 57.1 |
| **2014^3^** | | |
| **January** | ‒9.2 | 49.6 |
| **February** | ‒10.2 | 49.5 |
| **March** | ‒5.1 | 25.3 |
| **April** | +6.3 | 76.2 |
| **May** | +13.8 | 81.8 |
| **June** | +19.7 | 96.2 |
| **July** | +18.8 | 109.7 |
| **August** | +19.3 | 43.8 |
| **September** | +16.0 | 165.0 |

^1^ **MAT**: Mean Air Temperature (data from Environment Canada, 2015)

^2^ **MTP**: Monthly Total Precipitation (data from Environment Canada, 2015)

^3^Data from London CS station (43°02'00.000" N, 81°09'00.000" W)
