# Supplementary material for: Nitrogen isotopes suggest a change in nitrogen dynamics between the Late Pleistocene and modern time in Yukon, Canada
Source: PLoS One. 2018 Feb 15;13(2):e0192713. doi: 10.1371/journal.pone.0192713 (PMC5813965; doi:10.1371/journal.pone.0192713)
Supplement: S3 Table — (DOCX) [file pone.0192713.s003.docx]

**S3 Table:** FTIR-CI and C/P for fossil and modern bone bioapatite.

| **Sample ID** | **Site** | **CI** | **C/P** |
| --- | --- | --- | --- |
|  | **Fossil Bone** |  |  |
| **IC-9-2** | **IC** | 2.5 | 0.7 |
| **IC-9-1** | **IC** | 2.5 | 0.9 |
| **IC-19** | **IC** | 2.5 | 0.7 |
| **GC-GZ-3-1** | **GC** | 2.5 | 0.7 |
| **IC-14** | **IC** | 2.4 | 0.6 |
| **QC-4** | **QC** | 2.8 | 0.6 |
| **LB-GZ-1** | **LB** | 2.7 | 0.6 |
| **IC-3** | **IC** | 2.8 | 0.6 |
|  | **Modern Bone** |  |  |
| **M-1-female** | **WH** | 2.6 | 0.7 |
| **M-2-male** | **WH** | 2.5 | 0.6 |
| **M-3-male** | **WH** | 2.5 | 0.7 |
| **M-5-male** | **WH** | 2.5 | 0.7 |
| **M-6-male** | **WH** | 2.6 | 0.6 |
| **M-7-male** | **WH** | 2.5 | 0.7 |
| **M-8-female** | **WH** | 2.5 | 0.8 |
| **M-9-male** | **WH** | 2.5 | 0.7 |
| **M-10-male** | **WH** | 2.5 | 0.7 |
| **M-11-male** | **WH** | 2.7 | 0.6 |
| **M-12-male** | **WH** | 2.4 | 0.7 |
| **M-14** | **KL** | 2.4 | 0.7 |
| **M-15** | **KL** | 2.6 | 0.6 |
| **M-16** | **KL** | 2.6 | 0.5 |

WH: Whitehorse, Erik Nielsen International airport.

KL: Eastern shoreline of Kluane Lake.

IC: Independence Creek; GC: Glacier Creek.

QC: Quartz Creek; LB: Little Blanche Creek.
