# Supplementary figures and images for: Nitrogen isotopes suggest a change in nitrogen dynamics between the Late Pleistocene and modern time in Yukon, Canada
Source: PLoS One. 2018 Feb 15;13(2):e0192713. doi: 10.1371/journal.pone.0192713 (PMC5813965; doi:10.1371/journal.pone.0192713)

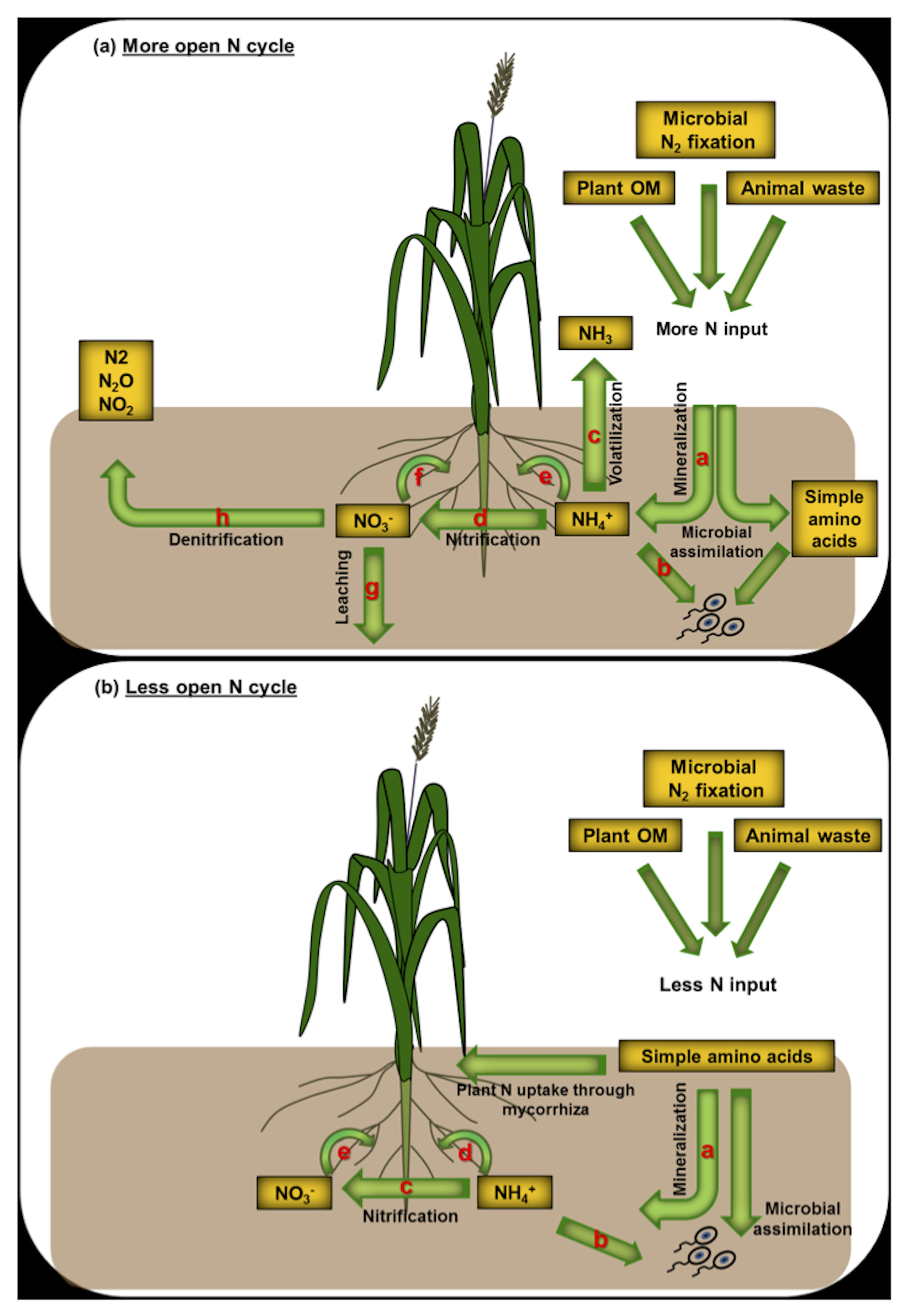

Supplement: S1 Fig — Asimplified model for the “openness” of the N cycle in ecosystems with high (a) and low (b) N availability (from Tahmasebi et al. [9]). (a): (a) N mineralization: Conversion of organic N to NH4+ (ε = 0–5 ‰); (b) Microbial assimilation: incorporation of NH4+ into microbial biomass (ε = 14–20 ‰); (c) NH3 volatilization: conversion of NH4+(aq) to NH3(g) (ε = 40–60 ‰); (d) Nitrification: conversion of NH4+ to NO3- (ε = 15–35 ‰); (e) Plant uptake and assimilation of NH4+ (ε = 9–18 ‰); (f) Plant uptake and assimilation of NO3- (ε = 0–19 ‰); (g) NO3- leaching (ε = 0–1 ‰); (h) Denitrification: conversion of NO3- to N2O, N2 and NO2 (ε = 28–33 ‰).(b): (a) N mineralization: Conversion of organic N to NH4+ (ε = 0–5 ‰); (b) Microbial assimilation: incorporation of NH4+ into microbial biomass (ε = 14–20 ‰); (c) Nitrification: conversion of NH4+ to NO3- (ε = 15–35 ‰); (d) Plant uptake and assimilation of NH4+ (ε = 9–18 ‰); (e) Plant uptake and assimilation of NO3- (ε = 0–19 ‰). Values of ε are from Robinson [29] and Houlton and Bai [105]). (TIF) [file pone.0192713.s004.tif]
