# Supplementary material for: Nitrogen isotopes suggest a change in nitrogen dynamics between the Late Pleistocene and modern time in Yukon, Canada
Source: PLoS One. 2018 Feb 15;13(2):e0192713. doi: 10.1371/journal.pone.0192713 (PMC5813965; doi:10.1371/journal.pone.0192713)
Supplement: S1 Text — Table A: Loess characteristics. Table B: Loess mineralogy based on XRD. (DOCX) [file pone.0192713.s005.docx]

**S1 Text**

**Loess Chemical and Physical Properties:**

As shown in Table S3-1, all samples are dominated by silt (> 57 wt. %, avg. 69.5 ± 6.7 wt. %), with average clay and sand contents of 17.2 wt. % and 13.4 wt. %, respectively, and classified as Silty Loam. Organic matter (OM) content ranges from 5.0 to 20.4 wt. %, and there is a strong correlation between organic carbon (OC; 1.8-9.5 wt. %) and OM contents (loss on ignition (LOI _550_)) (R = 0.998, *p*-value = 0.000). Loess pH (avg. 7.6 ± 0.3) indicates neutral to alkaline conditions. Bulk loess mineralogy, based on X-ray diffraction (XRD), comprises mainly quartz, feldspar and calcite; amphibole, phyllosilicates (mica/illite; kaolinite/septechlorite) and dolomite are detectable in a few samples (Table S3-2).

**Table A:** Loess characteristics.

| **Sample ID** | **pH** | **Texture** | **Sand** | **Silt** | **Clay** | **OM** | **OC** | **TC** | **TN** | **Atomic OC/TN** |
| --- | --- | --- | --- | --- | --- | --- | --- | --- | --- | --- |
|  |  |  | **wt. %** | | | | | | |  |
| **QC-2** | 7.6 | SiL | 1.6 | 78.0 | 20.4 | 5.8 | 2.0 | 2.4 | 0.2 | 12.1 |
| **QC-3** | 7.3 | SiL | 29.6 | 57.6 | 12.8 | 12.2 | 5.0 | 5.2 | 0.4 | 13.9 |
| **QC-4** | 7.1 | SiL | 11.6 | 67.6 | 20.8 | 20.4 | 9.5 | 9.0 | 0.6 | 17.6 |
| **QC-5** | 7.9 | SiL | 13.6 | 71.6 | 14.8 | 5.4 | 1.8 | 2.3 | 0.2 | 10.8 |
| **QC-6** | 7.8 | SiL | 7.6 | 75.6 | 16.8 | 5.4 | 1.9 | 2.3 | 0.2 | 11.4 |
| **QC-7** | 7.8 | SiL | 9.6 | 73.6 | 16.8 | 6.6 | 2.4 | 2.7 | 0.2 | 11.7 |
| **QC-8** | 7.8 | SiL | 15.6 | 67.6 | 16.8 | 6.0 | 1.9 | 2.3 | 0.2 | 11.7 |
| **IC-9** | 7.5 | SiL | 17.6 | 64.0 | 18.4 | 5.0 | 1.9 | 2.2 | 0.2 | 12.5 |

OM: Organic Matter; OC: Organic Carbon; TC: Total Carbon; TN: Total Nitrogen; SiL: Silty Loam

**Table B:** Loess mineralogy based on XRD

| **Mineral/ d-space (nm)** | **Quartz** | **Feldspar** | **Calcite** | **0.71^a^** | **Amphibole** | **1.01^b^** | **Dolomite** |
| --- | --- | --- | --- | --- | --- | --- | --- |
| **Site ID** | **wt. %** | | | | | | |
| **QC-2** | 88 | 8 | < 5 | < 5 | - | - | - |
| **QC-3** | 75 | 20 | - | < 5 | - | - | - |
| **QC-4** | 83 | 11 | - | 6 | - | - | - |
| **QC-5** | 79 | 7 | < 5 | 6 | - | - | 7 |
| **QC-6** | 76 | 13 | < 5 | < 5 | < 5 | < 5 | - |
| **QC-7** | 68 | 17 | < 5 | 7 | < 5 | < 5 | - |
| **QC-8** | 79 | 9 | 5.0 | < 5 | < 5 | < 5 | - |
| **IC-9** | 86 | 11 | - | - | - | < 5 | - |

^a^Septachlorite/Kaolinite

^b^Mica/Illite
