# Supplementary material for: Nitrogen isotopes suggest a change in nitrogen dynamics between the Late Pleistocene and modern time in Yukon, Canada
Source: PLoS One. 2018 Feb 15;13(2):e0192713. doi: 10.1371/journal.pone.0192713 (PMC5813965; doi:10.1371/journal.pone.0192713)
Supplement: S1 File — Table A: δ13C and δ15N for replicate analyses from the decomposition experiment. Table B: C and N contents for replicate analyses from the decomposition experiment. Table C: Atomic C/N of plant detritus during decomposition. (DOCX) [file pone.0192713.s006.docx]

**Table A:** *δ*^13^C and *δ*^15^N for replicate analyses from the decomposition experiment.

|  | ***δ*^13^C (‰, VPDB)** | | | | | | ***δ*^15^N (‰, AIR)** | | | | | |
| --- | --- | --- | --- | --- | --- | --- | --- | --- | --- | --- | --- | --- |
|  | **Buried** | | | **Not buried** | | | **Buried** | | | **Not buried** | | |
|  | **Day 1** | | | | | | | | | | | |
| **Plant ID** | **R1** | **R2** | **R3** | **R1** | **R2** | **R3** | **R1** | **R2** | **R3** | **R1** | **R2** | **R3** |
| ***E. trachycaulus*** | ‒27.7 | ‒27.7 | ‒27.8 | ‒27.7 | ‒27.7 | ‒27.8 | +1.0 | ‒2.1 | ‒1.8 | +1.0 | ‒2.1 | ‒1.8 |
| ***C. purpurascens*** | ‒27.3 | ‒27.6 | ‒27.5 | ‒27.3 | ‒27.6 | ‒27.5 | ‒0.3 | ‒0.9 | ‒1.2 | ‒0.3 | ‒0.9 | ‒1.2 |
| ***P. glauca*** | ‒26.4 | ‒25.6 | ‒24.9 | ‒26.4 | ‒25.7 | ‒24.9 | +0.6 | 0.5 | +0.8 | +0.6 | +0.5 | +0.8 |
| ***F. altaica*** | ‒27.6 | ‒27.6 | ‒27.6 | ‒27.6 | ‒27.6 | ‒27.6 | ‒0.8 | ‒6.3 | ‒7.0 | ‒0.8 | ‒6.3 | ‒7.0 |
| ***A. frigida*** | ‒30.1 | ‒29.0 | ‒29.3 | ‒30.1 | ‒29.0 | ‒29.3 | ‒3.6 | ‒1.3 | ‒2.4 | ‒3.6 | ‒1.3 | ‒2.4 |
| ***E. spicatus*** | ‒27.0 | ‒27.4 | ‒26.9 | ‒27.0 | ‒27.4 | ‒26.9 | ‒1.9 | ‒2.8 | ‒4.6 | ‒1.9 | ‒2.8 | ‒4.6 |
|  | **Day 164** | | | | | | | | | | | |
| ***E. trachycaulus*** | ‒28.1 | ‒26.7 | ‒27.6 | ‒28.3 | ‒28.7 | ‒28.2 | +6.8 | +3.1 | +5.8 | ‒0.8 | ‒1.0 | +1.2 |
| ***C. purpurascens*** | ‒24.5 | ‒24.4 | ‒24.3 | ‒25.0 | ‒25.1 | ‒24.6 | +3.6 | +2.4 | +3.5 | ‒1.9 | ‒1.6 | ‒0.3 |
| ***P. glauca*** | ‒26.1 | ‒26.7 | ‒26.3 | ‒26.4 | ‒26.8 | ‒26.8 | 0.0 | +1.7 | 2.0 | ‒1.0 | ‒1.3 | ‒0.5 |
| ***F. altaica*** | ‒27.1 | ‒27.5 | ‒27.2 | ‒28.0 | ‒27.5 | ‒27.6 | +5.2 | +0.5 | 5.3 | ‒1.7 | +0.2 | +1.0 |
| ***A. frigida*** | ‒30.8 | ‒30.5 | ‒30.8 | ‒29.3 | ‒29.6 | ‒29.3 | ‒0.5 | ‒1.1 | ‒0.8 | ‒2.2 | ‒2.0 | ‒1.7 |
| ***E. spicatus*** | ‒27.8 | ‒27.8 | ‒26.6 | ‒27.2 | ‒26.9 | ‒27.1 | +4.2 | +4.0 | +3.6 | ‒3.0 | ‒2.4 | ‒0.2 |
|  | | | | | | | | | | | | |
|  | | | | | | | | | | | | |
| **Table A: Cont’d.** | | | | | | | | | | | | |
|  | ***δ*^13^C (‰, VPDB)** | | | | | | ***δ*^15^N (‰, AIR)** | | | | | |
|  | **Buried** | | | **Not buried** | | | **Buried** | | | **Not buried** | | |
|  | **Day 253** | | | | | | | | | | | |
| **Plant ID** | **R1** | **R2** | **R3** | **R1** | **R2** | **R3** | **R1** | **R2** | **R3** | **R1** | **R2** | **R3** |
| ***E. trachycaulus*** | ‒29.2 | ‒28.0 | ‒28.7 | ‒27.5 | ‒28.4 | ‒27.0 | +3.9 | +5.1 | +4.4 | ‒1.8 | ‒0.8 | ‒0.8 |
| ***C. purpurascens*** | ‒25.4 | ‒23.8 | ‒24.7 | ‒25.6 | ‒24.8 | ‒24.9 | +3.4 | +2.4 | +3.0 | +0.7 | ‒5.0 | +0.2 |
| ***P. glauca*** | ‒26.4 | ‒27.1 | ‒26.7 | ‒26.4 | ‒27.5 | ‒26.8 | +2.1 | +3.7 | +2.1 | ‒3.4 | ‒2.6 | ‒1.5 |
| ***F. altaica*** | ‒28.2 | ‒28.0 | ‒27.7 | ‒27.2 | ‒27.6 | ‒27.5 | +6.8 | +7.1 | +5.5 | ‒4.2 | ‒0.1 | +1.3 |
| ***A. frigida*** | ‒29.2 | ‒30.5 | ‒30.9 | ‒29.6 | ‒29.5 | ‒30.0 | +0.7 | +1.3 | +0.8 | +1.9 | +0.3 | ‒1.8 |
| ***P. spicata*** | ‒28.4 | ‒28.1 | ‒25.4 | ‒27.2 | ‒26.8 | ‒28.7 | +0.5 | +6.1 | +6.3 | ‒5.2 | ‒2.7 | ‒2.2 |
|  | **Day 317** | | | | | | | | | | | |
| ***E. trachycaulus*** | ‒28.7 | ‒28.1 | ‒27.0 | ‒27.9 | ‒27.0 | ‒27.4 | +2.8 | +3.3 | +0.9 | ‒3.8 | ‒3.2 | ‒1.6 |
| ***C. purpurascens*** | ‒25.8 | ‒26.1 | ‒24.7 | ‒24.0 | ‒25.9 | ‒24.5 | +2.2 | +3.1 | +3.4 | ‒2.3 | ‒2.0 | ‒0.7 |
| ***P. glauca*** | ‒27.9 | ‒27.0 | ‒26.8 | ‒27.1 | ‒27.2 | ‒27.0 | +3.0 | +3.1 | +3.1 | ‒3.8 | ‒2.6 | ‒2.9 |
| ***F. altaica*** | ‒27.6 | ‒26.6 | ‒28.1 | ‒25.4 | ‒27.3 | ‒27.3 | +5.0 | +5.6 | +5.1 | ‒1.6 | ‒1.6 | ‒1.8 |
| ***A. frigida*** | ‒30.6 | ‒31.1 | ‒30.4 | ‒29.4 | ‒29.5 | ‒28.8 | ‒0.1 | +0.5 | +0.3 | ‒1.3 | ‒1.5 | ‒0.9 |
| ***E. spicatus*** | ‒27.3 | ‒26.8 | ‒27.6 | ‒27.5 | ‒28.0 | ‒26.9 | +3.7 | +4.2 | +4.4 | ‒5.4 | ‒2.9 | ‒4.3 |

R: Replicate

**Table B:** C and N contents for replicate analyses from the decomposition experiment.

|  | **C (wt. %)** | | | | | | **N (wt. %)** | | | | | |
| --- | --- | --- | --- | --- | --- | --- | --- | --- | --- | --- | --- | --- |
|  | **Buried** | | | **Not buried** | | | **Buried** | | | **Not buried** | | |
|  | **Day 1** | | | | | | | | | | | |
| **Plant ID** | **R1** | **R2** | **R3** | **R1** | **R2** | **R3** | **R1** | **R2** | **R3** | **R1** | **R2** | **R3** |
| ***E. trachycaulus*** | 42.9 | 41.9 | 44.3 | 42.9 | 41.9 | 44.3 | 0.3 | 1.0 | 0.8 | 0.3 | 1.0 | 0.8 |
| ***C. purpurascens*** | 39.1 | 41.0 | 43.0 | 39.1 | 41.0 | 43.0 | 0.6 | 0.7 | 0.5 | 0.6 | 0.7 | 0.5 |
| ***P. glauca*** | 41.0 | 41.4 | 40.6 | 41.0 | 41.4 | 40.6 | 0.9 | 1.5 | 1.3 | 0.9 | 1.5 | 1.3 |
| ***F. altaica*** | 39.1 | 42.6 | 41.2 | 39.1 | 42.6 | 41.2 | 0.3 | 0.9 | 0.9 | 0.3 | 0.9 | 0.9 |
| ***A. frigida*** | 44.0 | 45.9 | 44.8 | 44.0 | 45.9 | 44.8 | 1.3 | 1.4 | 1.5 | 1.3 | 1.4 | 1.5 |
| ***E. spicatus*** | 42.4 | 41.7 | 44.9 | 42.4 | 41.7 | 44.9 | 0.5 | 0.8 | 0.2 | 0.5 | 0.8 | 0.2 |
|  | **Day 164** | | | | | | | | | | | |
| ***E. trachycaulus*** | 43.0 | 39.0 | 40.6 | 40.6 | 43.2 | 40.4 | 0.5 | 0.6 | 1.1 | 0.4 | 0.4 | 1.1 |
| ***C. purpurascens*** | 40.8 | 40.0 | 37.0 | 39.9 | 37.5 | 37.9 | 0.7 | 0.8 | 0.8 | 0.5 | 0.6 | 0.6 |
| ***P. glauca*** | 40.2 | 38.9 | 40.6 | 42.3 | 39.4 | 40.0 | 1.0 | 0.9 | 0.8 | 0.7 | 0.9 | 1.1 |
| ***F. altaica*** | 28.2 | 38.2 | 34.3 | 37.8 | 34.1 | 32.1 | 0.8 | 0.5 | 0.7 | 0.4 | 0.6 | 0.7 |
| ***A. frigida*** | 48.2 | 46.5 | 46.9 | 48.9 | 45.2 | 44.7 | 1.1 | 1.1 | 0.9 | 1.2 | 1.3 | 1.2 |
| ***E. spicatus*** | 42.7 | 39.2 | 41.9 | 39.3 | 41.4 | 39.9 | 0.7 | 0.9 | 0.9 | 0.6 | 0.6 | 0.7 |
|  |  |  |  |  |  |  |  |  |  |  |  |  |
|  |  |  |  |  |  |  |  |  |  |  |  |  |
| **Table B: Cont’d.** | | | | | | | | | | | |  |
|  | **C (wt. %)** | | | | | | **N (wt. %)** | | | | | |
|  | **Buried** | | | **Not buried** | | | **Buried** | | | **Not buried** | | |
|  | **Day 253** | | | | | | | | | | | |
| **Plant ID** | **R1** | **R2** | **R3** | **R1** | **R2** | **R3** | **R1** | **R2** | **R3** | **R1** | **R2** | **R3** |
| ***E. trachycaulus*** | 42.1 | 31.5 | 38.9 | 41.8 | 44.5 | 32.4 | 1.3 | 1.3 | 1.2 | 0.5 | 0.6 | 1.0 |
| ***C. purpurascens*** | 37.4 | 34.6 | 30.1 | 34.7 | 43.8 | 35.6 | 1.3 | 0.9 | 1.5 | 0.9 | 0.4 | 0.7 |
| ***P. glauca*** | 24.6 | 30.5 | 29.4 | 38.8 | 35.9 | 28.6 | 1.2 | 1.5 | 1.5 | 0.8 | 0.8 | 1.0 |
| ***F. altaica*** | 35.8 | 28.0 | 31.6 | 42.4 | 36.0 | 24.6 | 1.7 | 1.3 | 1.0 | 0.3 | 0.6 | 0.8 |
| ***A. frigida*** | 40.8 | 44.0 | 42.8 | 46.3 | 49.6 | 42.5 | 2.2 | 1.5 | 1.6 | 1.2 | 1.0 | 1.1 |
| ***E. spicatus*** | 34.2 | 40.8 | 31.6 | 40.4 | 40.0 | 37.0 | 0.8 | 0.5 | 1.4 | 0.4 | 0.5 | 0.6 |
|  | **Day 317** | | | | | | | | | | | |
| ***E. trachycaulus*** | 41.9 | 35.9 | 35.7 | 38.8 | 37.3 | 39.2 | 1.2 | 1.4 | 1.6 | 0.5 | 0.5 | 0.6 |
| ***C. purpurascens*** | 31.1 | 39.4 | 38.0 | 34.1 | 29.3 | 34.7 | 1.2 | 1.1 | 1.2 | 0.7 | 0.9 | 0.8 |
| ***P. glauca*** | 29.1 | 30.3 | 28.5 | 29.5 | 30.9 | 32.2 | 1.5 | 1.6 | 1.5 | 0.9 | 1.0 | 0.9 |
| ***F. altaica*** | 36.0 | 31.0 | 29.6 | 24.1 | 28.4 | 38.5 | 1.1 | 1.0 | 1.3 | 0.7 | 0.7 | 0.5 |
| ***A. frigida*** | 45.8 | 43.1 | 39.1 | 45.6 | 44.8 | 43.0 | 1.1 | 1.4 | 1.3 | 1.3 | 1.2 | 1.2 |
| ***E. spicatus*** | 29.3 | 23.2 | 25.8 | 39.1 | 36.4 | 37.0 | 1.2 | 1.0 | 1.1 | 0.5 | 0.7 | 0.6 |

R: Replicate

**Table C:** Atomic C/N of plant detritus during

decomposition.

| **Plant** | **Time** | **Atomic C/N** | |
| --- | --- | --- | --- |
| **species** | **(days)** | **Buried** | **Not buried** |
| ***E. trachycaulus*** | **1** | 71.0 | 71.0 |
|  | **164** | 65.0 | 72.7 |
|  | **253** | 34.6 | 66.2 |
|  | **317** | 32.1 | 83.0 |
| ***C. purpurascens*** | **1** | 81.5 | 81.5 |
|  | **164** | 58.7 | 77.7 |
|  | **253** | 32.0 | 67.2 |
|  | **317** | 35.2 | 48.3 |
| ***P. glauca*** | **1** | 38.8 | 38.8 |
|  | **164** | 51.7 | 54.6 |
|  | **253** | 23.7 | 49.2 |
|  | **317** | 22.5 | 39.0 |
| ***F. altaica*** | **1** | 69.6 | 69.6 |
|  | **164** | 61.2 | 68.9 |
|  | **253** | 27.7 | 72.3 |
|  | **317** | 33.5 | 53.0 |
| ***A. frigida*** | **1** | 36.9 | 36.9 |
|  | **164** | 55.4 | 43.7 |
|  | **253** | 28.3 | 48.6 |
|  | **317** | 39.6 | 41.7 |
| ***E. spicatus*** | **1** | 105.2 | 105.2 |
|  | **164** | 58.7 | 75.6 |
|  | **253** | 46.1 | 88.3 |
|  | **317** | 27.4 | 72.5 |
