# Supplementary material for: Nitrogen isotopes suggest a change in nitrogen dynamics between the Late Pleistocene and modern time in Yukon, Canada
Source: PLoS One. 2018 Feb 15;13(2):e0192713. doi: 10.1371/journal.pone.0192713 (PMC5813965; doi:10.1371/journal.pone.0192713)
Supplement: S2 File — Table A: Benferroni post hoc test for differences in average δ13Clitter between Day 1 and Days 164, 253 and 317 for samples showing significant time effect. Table B: Benferroni post hoc test for differences in average δ15Nlitter between Day 1 and Days 164, 253 and 317 for samples showing significant time effect. (DOCX) [file pone.0192713.s007.docx]

**Table A:** Benferroni *post hoc* test for differences in average *δ*^13^C_litter_ between Day 1 and Days 164, 253 and 317 for samples showing significant time effect.

| **Plant ID** | **B-1 to B-164** | **B-1 to B-253** | **B-1 to B-317** |
| --- | --- | --- | --- |
|  | ***δ*^13^C (‰, VPDB)** | | |
| ***C. purpurascens*** | **0.010** | 0.203 | 0.256 |

B: Buried 1: Day 1 253: Day 253 317: Day 317

Values in boldface are statistically significant (*p* ≤ 0.05).

**Table B:** Benferroni *post hoc* test for differences in average *δ*^15^N_litter_ between Day 1 and Days 164, 253 and 317 for samples showing significant time effect.

| **Plant ID** | **B-1 to**  **B-164** | **B-1 to**  **B-253** | **B-1 to**  **B-317** | **NB-1 to**  **NB-164** | **NB-1 to**  **NB-253** | **NB-1 to**  **NB-317** |
| --- | --- | --- | --- | --- | --- | --- |
|  | ***δ*^15^N (‰, AIR)** | | | | | |
| ***E. trachycaulus*** | 0.080 | 0.317 | 0.551 | **-** | **-** | **-** |
| ***C. purpurascens*** | 0.073 | **0.034** | 0.173 | **-** | **-** | **-** |
| ***P. glauca*** | 1.000 | 0.513 | **0.015** | **-** | **-** | **-** |
|  | **-** |  | **-** | **0.031** | 0.145 | 0.066 |
| ***F. altaica*** | 0.317 | 0.149 | 0.240 | **-** | **-** | **-** |
| ***A. frigida*** | 1.000 | 0.136 | 0.210 | **-** | **-** | **-** |

B: Buried NB: Not Buried 1: Day 1 253: Day 253 317: Day 317

Values in boldface are statistically significant (*p* ≤ 0.05).
